# The Role of cGAS-STING in Age-Related Diseases from Mechanisms to Therapies

**DOI:** 10.14336/AD.2023.0117

**Published:** 2023-08-01

**Authors:** Weitao Zheng, Dechao Feng, Xingyu Xiong, Xinyang Liao, Sheng Wang, Hang Xu, Weizhen Le, Qiang Wei, Lu Yang

**Affiliations:** Department of Urology, Institute of Urology, West China Hospital, Sichuan University, Chengdu, Sichuan, China.

**Keywords:** cGAS-STING, age-related diseases, cardiovascular diseases, neurological disorders, neoplasms, mechanisms, therapies

## Abstract

With aging, the incidence of age-related diseases increases. Hence, age-related diseases are inevitable. However, the mechanisms by which aging leads to the onset and progression of age-related diseases remain unclear. It has been reported that inflammation is closely associated with age-related diseases and that the cGAS-STING signaling pathway, which can sense the aberrant presence of cytosolic DNA during aging and induce an inflammatory response, is an important mediator of inflammation in age-related diseases. With a better understanding of the structure and molecular biology of the cGAS-STING signaling axis, numerous selective inhibitors and agonists targeting the cGAS-STING pathway in human age-related diseases have been developed to modulate inflammatory responses. Here, we provide a narrative review of the activity of the cGAS-STING pathway in age-related diseases and discuss its general mechanisms in the onset and progression of age-related diseases. In addition, we outline treatments targeting the cGAS-STING pathway, which may constitute a potential therapeutic alternative for age-related diseases.

Aging is an intrinsic feature of mammals associated with a gradual functional decline over time, leading to the progressive loss of physiological integrity and deterioration of multiple organ systems, a critical cause of many age-related diseases [1]. Age-related diseases are diseases that occur at quadratically increased incidence rates in the adult population with advancing age [2]. Although the biological causes of the aging process remain unknown, there are some common cellular and molecular traits known as aging hallmarks [3]. DNA integrity and stability are continuously affected by exogenous and endogenous agents throughout life, leading to the accumulation of genetic damage and genomic instability [4, 5]. Although genomic stability systems exist, the genetic damage generated during the aging process will exceed their limited repair capacity. This will eventually lead to the accumulation of excess genetic damage in cells and aging. Genomic stability systems include mechanisms that repair most of the damage to nuclear DNA, ensure the integrity of mitochondrial DNA (mtDNA), and maintain the length and functionality of telomeres [6-8]. Furthermore, the efficacy of the respiratory chain degenerates as cells and organisms age, resulting in mitochondrial dysfunction. Under normal conditions, cellular autophagy or mitophagy can eliminate aberrant cytosolic DNA via the action of nucleases in the cytosol and endolysosomal compartments. However, aging cells have autophagy or mitophagy defects, and the combination of mitochondrial dysfunction and autophagy or mitophagy defects may be responsible for several age-related diseases [9].

Genomic instability and mitochondrial dysfunction in aging are associated with the stochastic release of dsDNA from the nucleus and mitochondria into the cytoplasm [10, 11]. The aberrant presence of cytosolic dsDNA is recognized as a danger-associated molecular pattern (DAMP) by the DNA sensor cyclic guanosine monophosphate (GMP)-adenosine monophosphate (AMP) synthase (cGAS) [12]. The C-terminal part of human cGAS consists of DNA-binding sites (one primary site and two additional sites) that can bind to the sugar-phosphate backbone of DNA. The catalytic pocket of the enzyme rearranges, and the conformation of cGAS changes to provide optimal interaction with the substrates when DNA binds to the primary site. DNA binding to these two additional sites is important in the formation of the minimal active enzymatic unit that acts on GTP and ATP, inducing the synthesis of cyclic guanosine monophosphate-adenosine monophosphate (cGAMP) [13]. cGAMP functions as an endogenous second messenger and is detected by the stimulator of interferon (IFN) genes (STING) located in the endoplasmic reticulum (ER) membrane [14, 15]. When GAMP binds to STING, the STING conformation changes and STING is transferred from the ER to the Golgi apparatus through the ER-Golgi intermediate compartment (ERGIC), where it is activated [16]. Activated STING polymerizes and activates TANK-binding kinase 1 (TBK1). In addition, TBK1 recruits and phosphorylates IFN regulatory factor 3 (IRF3), resulting in its dimerization, nuclear translocation, and interaction with nuclear factor κB (NF-κB) to trigger the synthesis of IFN and inflammatory cytokines (Fig. 1) [13, 17].

**Figure 1.**
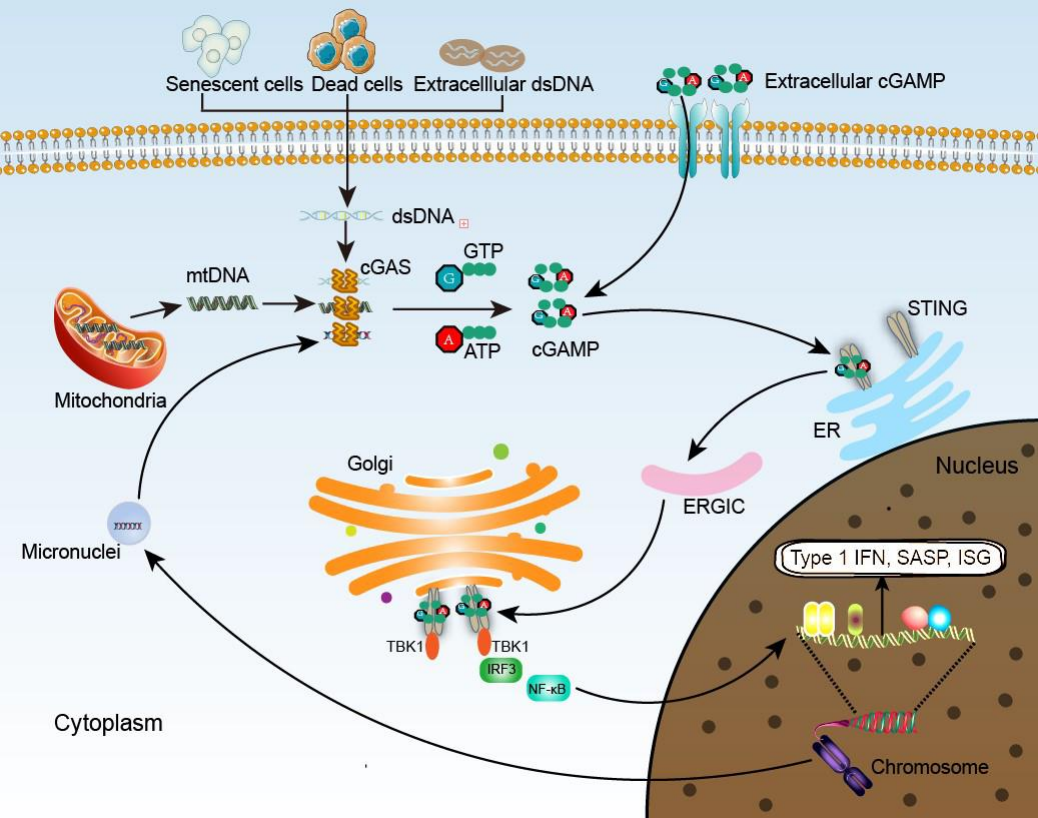
**Overview of the cGAS-STING pathway in age-related diseases**. cGAS is a cytosolic DNA sensor that detects dsDNA from senescent cells and dead cells, and some extracellular dsDNA, mtDNA and DNA in the micronuclei are also the main sources of cytosolic DNA. After combining with DNA, cGAS interacts with GTP and ATP, inducing the synthesis of cGAMP. In addition to intracellular synthesis, extracellular cGAMP can also enter the cell and acts as a second messenger, binding to STING located in the ER. Activated STING then transfers to the Golgi via ERGIC, where it can truly function, interacting with TBK1, IRF3, and NF-κB and mediating the production of type I IFN, SASP and ISG, promoting the inflammatory response and exerting biological effects.

Senescent cells accumulate with age. Although these cells undergo a stable cell cycle arrest and cannot duplicate, they can produce a complex secretome, including cytokines, chemokines, growth factors, and proteases. This is called the senescence-associated secretory phenotype (SASP)[18]. The functions of different SASP members differ greatly and are associated with autocrine and paracrine signaling, pro-tumorigenic and tumor-suppressive effects, and pro- and anti-inflammatory signaling [19]. The cGAS-STING pathway plays an indispensable role in cellular senescence, as cellular senescence induced by oxidative stress, DNA-damaging drugs, and oncogene activation is dependent on it [20, 21].

Senescent cells secrete SASP, such as IFN and interleukin (IL), in a cGAS-dependent manner, providing a critical paracrine signal to maintain cellular senescence. In addition, SASP can recruit immune cells to modulate the tissue immune microenvironment and monitor and eliminate tumor cells [22]. However, SASP is a double-edged sword; it can also promote tumorigenesis by inducing the proliferation, transformation, invasiveness, and metastasis of epithelial cells [23, 24].

Given the important roles of the cGAS-STING pathway in cellular senescence and age-related diseases, we critically evaluated the mechanisms of cGAS-STING in age-related diseases, as well as novel therapeutic approaches targeting this pathway.

## Cardiovascular diseases (CVDs)

**Figure 2.**
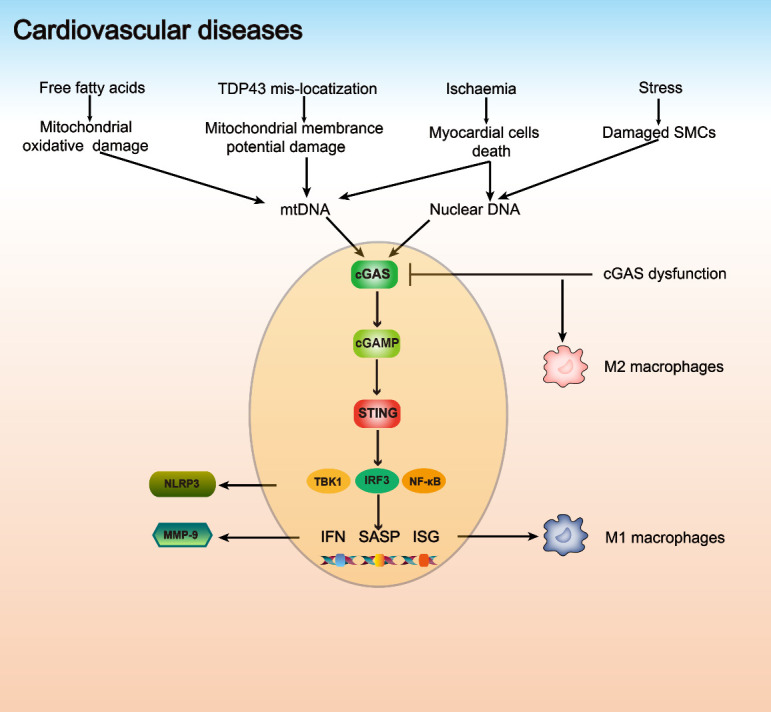
**The cGAS-STING pathway in cardiovascular diseases**. Free fatty acids can cause mitochondrial oxidative damage, and TDP43 mislocalization results in mitochondrial potential damage. This damage to mitochondria leads to the leakage of mtDNA into the cytoplasm. Damaged SMCs and myocardial cells release nuclear DNA; in addition, dead myocardial cells release mtDNA into the cytoplasm due to ischemia. This cytosolic dsDNA activates the cGAS-STING pathway and induces the production of NLRP3, MMP-9 and M1 macrophages (proinflammatory and antimicrobial), and inhibition of cGAS promotes the formation of M2 macrophages (healing, profibrotic, and anti-inflammatory).

CVDs are the leading cause of death, accounting for almost 31% of all deaths worldwide. In 2016, it was estimated that 17.9 million people died of CVDs [25]. Although epidemiological evidence illustrates that advanced age promotes CVDs, the complete mechanisms of how aging drives CVD progression remain unclear [26]. Recent studies have indicated that the cGAS-STING pathway plays an important role in CVDs (Fig. 2) [27].

### Cardiac hypertrophy

Myocyte enlargement induced by prolonged and abnormal hemodynamic stress is a characteristic of pathological cardiac hypertrophy, which usually presents with hypertension. The complex responses of fibrosis, capillary thinning, increased production of proinflammatory cytokines, cellular dysfunction, and maladaptive epigenetic changes in pathological cardiac hypertrophy are major predisposing factors for maladaptive cardiac remodeling, cardiac dysfunction, and heart failure [28, 29].

TBK1/IRF3 signaling is associated with mechanical overload-induced cardiac hypertrophy [30]. The expression of STING was found to be increased both in the pathological cardiac hypertrophy heart tissues of patients and mice and correlated with the upregulated type I IFNs. STING knockout or deletion in mice remarkably alleviates cardiac hypertrophy by reducing cardiomyocyte size, ameliorating cardiac function, and decreasing hypertrophic markers, inflammatory factors, and fibrosis. Furthermore, STING knockdown abrogated the factors secreted by cardiomyocytes in vitro, which are associated with collagen accumulation in cardiac fibroblasts. These findings demonstrate that the cGAS-STING pathway plays an important regulatory role in the pathogenesis of pathological cardiac hypertrophy [31].

Free fatty acids generated by injections of streptozotocin and a high-fat diet (HFD) in diabetes models can impair mitochondrial function. In addition, the leakage of mtDNA into the cytosol activates the cGAS-STING pathway in mice. Its activation subsequently promotes the formation of the nucleotide-binding oligomerization domain-like receptor pyrin domain containing 3 (NLRP3) inflammasome in the hearts of diabetic mice and the secretion of proinflammatory cytokines related to cardiac pyroptosis and chronic inflammation, ultimately promoting pathological cardiac hypertrophy [32]. HFD can induce cardiac dysfunction and promote maladaptive cardiac remodeling via chronic activation of the cGAS-STING signaling axis. Protein kinase B (Akt) and AMP-dependent protein kinase (AMPK) are closely related to cardiac energy metabolism and the maintenance of cardiac homeostasis. Double knockout of cardiac Akt isoform (Akt2) and AMPK accentuated the HFD-induced increases in heart weight, cardiac hypertrophy, myocardial apoptosis, cardiomyocyte contractile, and oxidative stress. Akt2-AMPK double ablation enhanced the HFD-induced activation of the cGAS-STING signaling pathway and promoted the production of proinflammatory cytokines. Cardiac function was improved, and myocardial hypertrophy was reduced after inhibiting cGAS or STING [33, 34]. The remarkable mitochondrial oxidative damage to cardiomyocytes in patients with chronic kidney disease (CKD) causes mtDNA leakage, which activates the cGAS-STING-NFκB pathway and NFκB-transactivated ornithine decarboxylase (ODC1)-putrescine metabolic flux, inducing cardiac hypertrophy. Inhibition of the myocardial mitochondria-STING-NFκB-ODC1 axis using genetic or pharmacological approaches significantly alleviated CKD-associated cardiac hypertrophy [35].

### Atherosclerosis (AS)

AS is a chronic inflammatory disease that affects the large- and medium-sized arteries. Early in AS, scavenging macrophages clear atherogenic lipoproteins and lead to the accumulation of intracellular lipids and the formation of foam cells. The residual lipid from the dead foam cells is deposited in the arteries along with the proliferation of the fibrous matrix and smooth muscle cells, forming the characteristic plaques of AS. Plaque rupture is the most common cause of coronary artery thrombosis and can result in heart attack and stroke [36-38]. Inflammation has been recognized as an important mediator of the onset and progression of AS [39].

The mislocalization of DNA/RNA binding protein transactive response DNA-binding protein 43 kDa (TDP43) in the cytoplasm impairs the mitochondrial membrane potential, causes oxidative stress, and induces the release of mtDNA into the cytoplasm. Increased TDP43 expression in both oxidized LDL (oxLDL)-treated macrophages and peripheral blood mononuclear cells from patients with coronary artery disease (CAD) can activate inflammation via NF-κB signaling in a cGAS-dependent manner; TDP43 knockout in macrophages significantly alleviated AS progression['\ 40]. Furthermore, it was recently demonstrated that stromal interacting molecule 1 (STIM1) regulates the cytosolic Ca^2+^ concentration and activates the cGAS-STING pathway, promoting vascular smooth muscle cell (VSMC) proliferation and apoptosis, resulting in fragile plaque formation and AS progression [41]. Mitochondrial damage in the VSMCs of CKD patients activates the cGAS-STING pathway and promotes the expression of type I IFN, leading to loss and thinning of the fibrous cap of VSMCs. This is responsible for the vulnerability of atherosclerotic plaques, accelerated atherosclerosis development, and the high morbidity rate of CVDs in patients with CKD [42].

### Myocardial infarction (MI)

MI is characterized by myocardial cell death caused by prolonged ischemia, which is a minor event in a lifelong chronic disease that could be the first manifestation of CAD, occurring during unstable periods of AS or recurring constantly in established MI patients. MI is a leading cause of global disability and death [43].

Massive myocardial cell death induced by ischemia during MI increases the release of nuclear DNA and mtDNA. The accumulated cytosolic DNA then stimulates the cGAS-STING pathway, inducing the production of IRF3 and its target prototypical cytokine type I IFNs (IFNα and IFNβ) transcripts. The IRF3-IFN axis was activated in different populations of IFN-inducible cells in MI mice. cGAS deficiency, genetic disruption of IRF3-dependent signaling, or antibody blockade of the type I IFN receptor in mice results in the downregulation of inflammatory cytokines and chemokines, alleviated inflammatory cell infiltration, and improved cardiac function and survival after MI [44]. Furthermore, selective small-molecule STING inhibitors, C178 and H-151, could reduce pathological remodeling, improve cardiac function, and alleviate heart failure after MI by disturbing the palmitoylation of STING. Cao et al. ligated the LAD coronary artery of mice to induce MI and found that cGAS-STING consistently and significantly increased the mRNA expression of IFN-stimulated genes (ISGs), including IRF7, IFN-induced protein with tetratricopeptide repeat 1 (IFIT1), IFIT3, chemokine (C-X-C motif) ligand 10 (CXCL10), and cluster of differentiation 14 (CD14). Importantly, the expression of STING increased without influencing the expression of major proinflammatory cytokines, such as IL1β, IL6, IL6, and tumor necrosis factor α (TNFα), by the inactivation of the cGAS-STING pathway at the level of transcript abundance [45]. M1-subtype macrophages (pro-inflammatory and antimicrobial) and M2-subtype macrophages (healing, profibrotic, and anti-inflammatory) are the major cell types in early MI [46, 47]. Cytosolic DNA induced the expression of the M1 markers inducible nitric oxide synthase (iNOS) and CXCL10 via the cGAS-STING pathway. However, cGAS signaling deficiency promotes the abundance of M2 macrophages, which can promote myofibroblast angiogenesis and transformation to enhance myocardial wound repair, alleviate pathological ventricular remodeling, and improve survival. cGAS-STING pathway activation in human heart failure samples was confirmed by the dynamic changes in cGAS and CXCL10, which were consistent with those observed in mice [45].

### Aortic aneurysm (AA) and dissection (AAD)

AA is the second most common aortic disease, characterized by permanent dilation of the thoracic and abdominal aortic regions compared to normal individuals[48]. Aortic dissection is characterized by true and false lumens of the aortic wall due to tears in the inner layer. Advanced age and vascular inflammation are two common risk factors for AA and dissection (AAD) [49]. The most important features of AAD are extracellular matrix (ECM) depletion and progressive loss of smooth muscle cells (SMCs) [50].

Significant DNA damage and leakage were observed in the aortic media and adventitia cells of patients with AAD, which resulted in the accumulation of cytosolic DNA and activation of the STING-TBK1-IRF3 pathway. This was confirmed by the upregulated expression and phosphorylation of STING, TBK1, and IRF3 in aortic tissues from patients with AAD. The STING-TBK1-IRF3 signaling pathway is critical in SMC necroptosis and apoptosis induced by ROS and cytosolic DNA. The DNA from damaged SMCs can activate the STING-TBK1-IRF3 signaling pathway in macrophages, inducing the production of matrix metalloproteinase (MMP)-9, and ultimately contributing to aortic degeneration and AAD formation. In the Sting ^gt/gt^ mouse model of sporadic AAD model, aortic enlargement and fiber disruption were alleviated, and the incidence of AAD was decreased in both the thoracic and abdominal aortic regions compared with the sporadic AAD wild-type mouse model. The TBK1 inhibitor amlexanox can significantly preserve the aortic structure and reduce aortic dilatation and the AAD incidence, improving survival and preventing the progression of AAD [51].

## Neurological disorders

**Figure 3.**
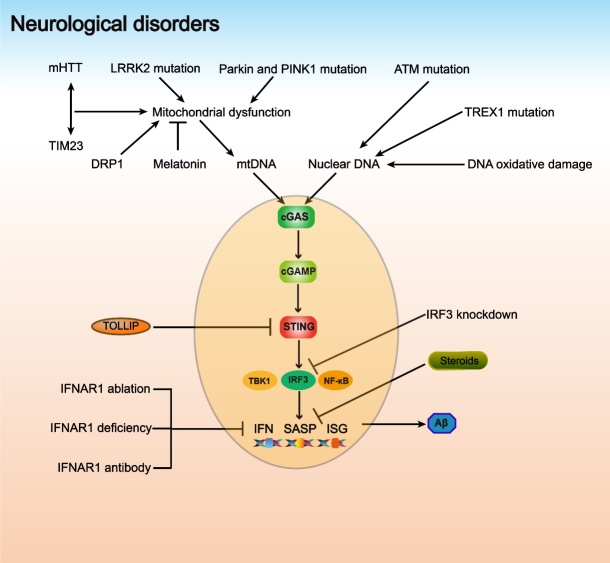
**The cGAS-STING pathway in neurological disorders**. mHTT has a high affinity for interacting with TIM23, the interaction of which damages the function of mitochondria. Furthermore, DRP1 and LRRK2 mutations and Parkin and PINK1 mutations also cause mitochondrial dysfunction, resulting in mtDNA leakage. Melatonin stabilizes mitochondria and inhibits mtDNA release. ATM mutation, TREX1 mutation and DNA oxidative damage contribute to the increase in nuclear DNA. Activation of the cGAS-STING pathway promotes Aβ-induced neurotoxicity. TOLLIP, steroids, IFNAR1 blockade (ablation, deficiency and antibody) and IRF3 knockdown inhibit the cGAS-STING pathway.

Aging is associated with several neurodegenerative diseases, and age-related brain inflammation, known as “inflammaging,” can lead to cognitive decline and deficits. The SASP phenotype induced by the cGAS-STING pathway plays an important role in neuroinflammation and age-related neurological disorders (Fig. 3) [52].

### Alzheimer’s disease (AD)

AD is the most common cause of dementia, affecting the daily activities of patients with AD. It is a major cause of dependence, disability, and mortality, and is recognized as a global public health priority. AD usually occurs in elderly individuals, resulting in insidious progressive problems in episodic memory. The representative pathological features of AD are extracellular accumulation of senile plaques containing amyloid-beta (Aβ), amyloid plaques, and neurofibrillary tangles (NFTs) containing hyperphosphorylated tau, accompanied by neuropil threads, dystrophic neurites, associated astrogliosis, microglial activation, and cerebral amyloid angiopathy [53].

Advanced age is the greatest risk factor for AD, mainly occurring in people over 65 years of age [54]. Neuroinflammation contributes to AD pathology, as confirmed by the enhanced microglial and astroglial activation, upregulated proinflammatory cytokine levels in the brains of patients with AD, and the decreased incidence of AD in individuals treated with nonsteroidal anti-inflammatory drugs. Type I IFN aggravates neuroinflammation and promotes AD development. Inhibition of IFN signaling within the aged brain partially rescues hippocampal neurogenesis and cognitive function and re-establishes IFN1-dependent choroid plexus activity during aging [55, 56]. The choroid plexus transcriptome demonstrated an overall upregulated expression of type I IFN response genes at all ages in AD mouse models [57]. Modulation of neuroinflammatory responses by inhibiting type I IFN signaling may provide new potential therapeutic targets in AD. Type I IFN receptor-1 (IFNAR1) is a highly specific transducer of type I IFN. Upon binding to type I IFN, IFNAR1 can trigger tyrosine phosphorylation of many proteins, including JAK, TYK2, and STAT, further regulating immune cell recruitment and inflammatory progression. Current research suggests that the removal of type 1 IFN signaling can reduce neuroinflammation and delay phenotypic progression. IFNAR1-deficient mice displayed a decrease in type I IFN and proinflammatory cytokines. Although astrocyte reactivity was enhanced, microglial proliferation around amyloid plaque deposition was attenuated. In addition, conditioned media from IFNAR1^-/-^ primary glia treated with Aβ1-42 were less toxic to primary cultured neurons [58]. The expression of IFNα in the brains of the APP/PS1 transgenic mouse model of AD was two-fold higher than that in control brains, and ablation of IFNAR1 and IRF3 or IRF7 knockdown protected cells against Aβ-induced neurotoxicity [59, 60]. Taken together, these findings indicate that the cGAS-STING-IFN pathway plays a critical role in the onset and development of AD, and that treatments targeting this pathway can alleviate IFN-related neuroinflammation and delay the progression of AD to some extent [61].

### Huntington’s disease (HD)

HD is a progressive neurodegenerative disease accompanied by motor, cognitive, and psychiatric disorders. A CAG repeat expansion with a polyglutamine strand of variable length confers direct toxicity to the mutant huntingtin (mHTT) protein, the main cause of HD[62]. HD can manifest throughout life, and the typical symptoms are chorea and dystonia, incoordination, cognitive decline, and behavioral difficulties, usually occurring in middle-aged individuals [63]. CAG repeats, but not the polyglutamine strand length, determine the age at HD onset[64]. Correia et al. conducted genome-wide association studies to discover genetic modifiers of HD and demonstrated that the age of HD onset was associated with oxidative DNA damage, DNA repair, and mitochondrial function pathways [65].

mHTT has a high affinity for translocase of the inner mitochondrial membrane 23 (TIM23), and their interaction can reduce the transportation of mitochondrial matrix proteins, altering the mitochondrial proteome and leading to the profound mitochondrial dysfunction documented in HD. mHtt can enhance the activity of mitochondrial fission GTPase dynamin-related protein-1 (DRP1) by binding to DRP1, resulting in excessive fragmentation and abnormal mitochondrial distribution. Subsequently, the increase in ROS causes mitochondrial damage and massive release of mtDNA, which triggers cGAS signaling and leads to the production of proinflammatory cytokines. Exploring new drugs that block the increase in ROS and the release of mtDNA may provide novel treatment options for aging and neurodegenerative diseases such as HD. Melatonin protects neurons from mHTT-mediated neurotoxicity. However, melatonin progressively decreases with age and in HD, and is a main cause of HD onset [66, 67]. mtDNA damage of the striatum and cerebral cortex in HD mice progressively increased and was eight times more frequent than nuclear DNA damage. An age-dependent increase in mtDNA damage contributes to mitochondrial dysfunction in HD and is an early biomarker of HD-associated neurodegeneration [68]. Cytosolic mtDNA in HD mice and mHTT-expressing cells stimulates the cGAS-STING-IRF3 pathway and subsequent production of proinflammatory cytokines. Evidence of cGAS-STING-IRF3 signaling pathway activation has also been reported in the early stage of human HD striatum, indicating that mtDNA release into the cytoplasm plays an important role in HD inflammation as an initial agent to activate the cGAS-STING-IRF3 signaling pathway [67]. Toll-interacting protein (TOLLIP) negatively regulates the Toll-like receptor (TLR) signaling pathway, which is associated with a variety of inflammatory diseases. TOLLIP is a stabilizer of STING and mediates the clearance of HD-linked polyQ protein aggregates, which can inhibit the STING-induced immune response by sequestering TOLLIP away from STING. The expression of TOLLIP decreases in the striatum of HD mice [69, 70].

### Parkinson’s disease (PD)

Selective neuronal loss in the substantia nigra and other brain areas is a characteristic of PD, the most common serious movement disorder and second most common neurodegenerative disease worldwide. Age is the strongest risk factor for PD. The typical syndromes of PD are rigidity, rest tremor, and bradykinesia [71, 72]. The pathological features of PD include damage to dopaminergic projections from the substantia nigra pars compacta to the caudate nucleus and putamen (striatum), and its pathological hallmarks are intraneuronal Lewy bodies and Lewy neurites [73]. Neuroinflammation is a critical cause of progressive PD. Activated microglia and upregulated proinflammatory cytokine expression were detected in both PD patients and animal models, and increased neuroinflammation damaged dopaminergic neuronal cells, resulting in PD [74, 75].

Mutations in the leucine-rich-repeat kinase 2 (LRRK2) gene in familial PD are recognized as the most common cause. Asymptomatic PD subjects with LRRK2 mutations have higher proinflammatory cytokine levels, which in turn increase LRRK2 kinase activity [76, 77]. Furthermore, LRRK2 maintains mitochondrial homeostasis, and LRRK2 deficiency in macrophages improves the basal levels of IFN. Oxidative stress mediated by purine metabolites and mitochondrial fission induced by DRP1 in LRRK2 knockout macrophages contributes to mtDNA leakage into the cytosol, which chronically activates the cGAS-STING pathway, and reduces DRP1 expression. Antioxidant treatment alleviates mitochondrial damage and decreases type I IFN expression in LRRK2 knockout macrophages [78]. Type I IFN signaling is upregulated both in the postmortem brains of PD patients and in a PD mouse model induced by 1-methyl-4-phenyl-1,2,3,6-tetrahydropyridine (MPTP). IFNAR1-deficient mice displayed decreased type I IFN signaling, proinflammatory response, and dopaminergic neuron loss. Monoclonal IFNAR1 antibody blockade treatment significantly upregulated striatal dopamine levels and improved behavior. This indicated that blocking IFNAR1 reduces MPTP-induced neuroinflammatory responses and dopaminergic cell death [79]. Parkin and PINK1 remove damaged mitochondria via mitophagy and mitigate STING-induced inflammation. Mutations in Parkin and PINK1 result in the accumulation of mtDNA mutations with age, the main cause of early onset PD. Blocking monoclonal IFNAR1 antibody treatment or STING deficiency rescued the inflammation induced by exhaustive exercise and mtDNA mutation, motor defects, and loss of dopaminergic neurons in aged Parkin-deficient mice [80].

### Aicardi-Goutières syndrome (AGS)

AGS is a recessive and progressive neurological disease associated with inflammation. The constitutive upregulation of type I IFN expression mediated by AGS-associated gene mutations is considered a major cause of AGS pathogenesis. Thus, ‘type I interferonopathy’ is also suggested to define the wider spectrum of diseases induced by mutations of AGS-associated genes [81, 82].

Aberrant activation of the cGAS-STING signal by self-DNA can lead to an abnormal innate immune response, which is the primary pathogenic factor in AGS and causes other severe autoimmune diseases. Three-prime repair exonuclease 1 (TREX1) dysfunction has been suggested to be associated with autoimmune diseases and inflammation. Mutations in TREX1, a 3’-DNA exonuclease that degrades cytoplasmic DNA, result in DNA degradation disorders. Furthermore, cytoplasmic chromatin DNA fragments contain DNA damage, and oxidative damage to DNA can inhibit the exonuclease activities of TREX1, collectively leading to the accumulation of cytosolic DNA [83, 84]. The increased cytosolic DNA subsequently provokes the cGAS-STING pathway and induces the production of type I IFN[85]. The upregulated expression of cGAMP in the inflamed hearts of TREX1-deficient mice confirmed the activation of cGAS in vivo. The mortality and pathology of TREX1-deficient mice with IFNAR deficiency significantly improved, indicating the important role of the cGAS-STING-IFN signaling axis in TREX1 mutation. Given the close relationship between AGS and TREX1 mutations, cGAS inhibitors may be a potential therapy for patients with AGS [86-88].

### Ataxia-telangiectasia (A-T)

A-T is a progressive neurodegenerative disorder characterized by immune dysfunction, predisposition to cancer, cerebellar degeneration, and telangiectasia. The underlying cause of A-T is mutations in the ataxia telangiectasia mutated (ATM) gene, which are important for dealing with DNA damage and maintaining genomic stability [89, 90]. More evidence indicates that chronic inflammation plays a critical role in the onset and development of A-T [91].

Increased numbers of micronuclei and nuclear shape abnormalities are observed in the olfactory neurosphere-derived cells (ONS) of A-T patients, and SASP genes, including IL1α, IL-6, and IL-8, are upregulated. Interfering with the expression of cGAS and STING with siRNAs decreased the abundance of SASP genes. In addition, A-T brain organoid senescence is reduced, and A-T neuropathology is improved by cGAS or STING inhibition [92]. Quek et al. constructed an ATM knockout rat model with paralysis and spinal cord atrophy, which was similar to older patients and milder forms instead of cerebellar atrophy. They found that the levels of cytosolic DNA dramatically increased in the neurons and glia of ATM-deficient rats. The accumulated DNA in the cytoplasm consequently activated the cGAS-STING signaling axis, accompanied by cytokine upregulation, formation of an inflammatory microenvironment, and constitutive activation of microglia, contributing to neuronal dysfunction and death. Short-term treatment with steroids, such as betamethasone, could significantly reduce the loss of motor neurons in the spine, presumably via its anti-inflammatory function [93].

## Neoplasms

**Figure 4.**
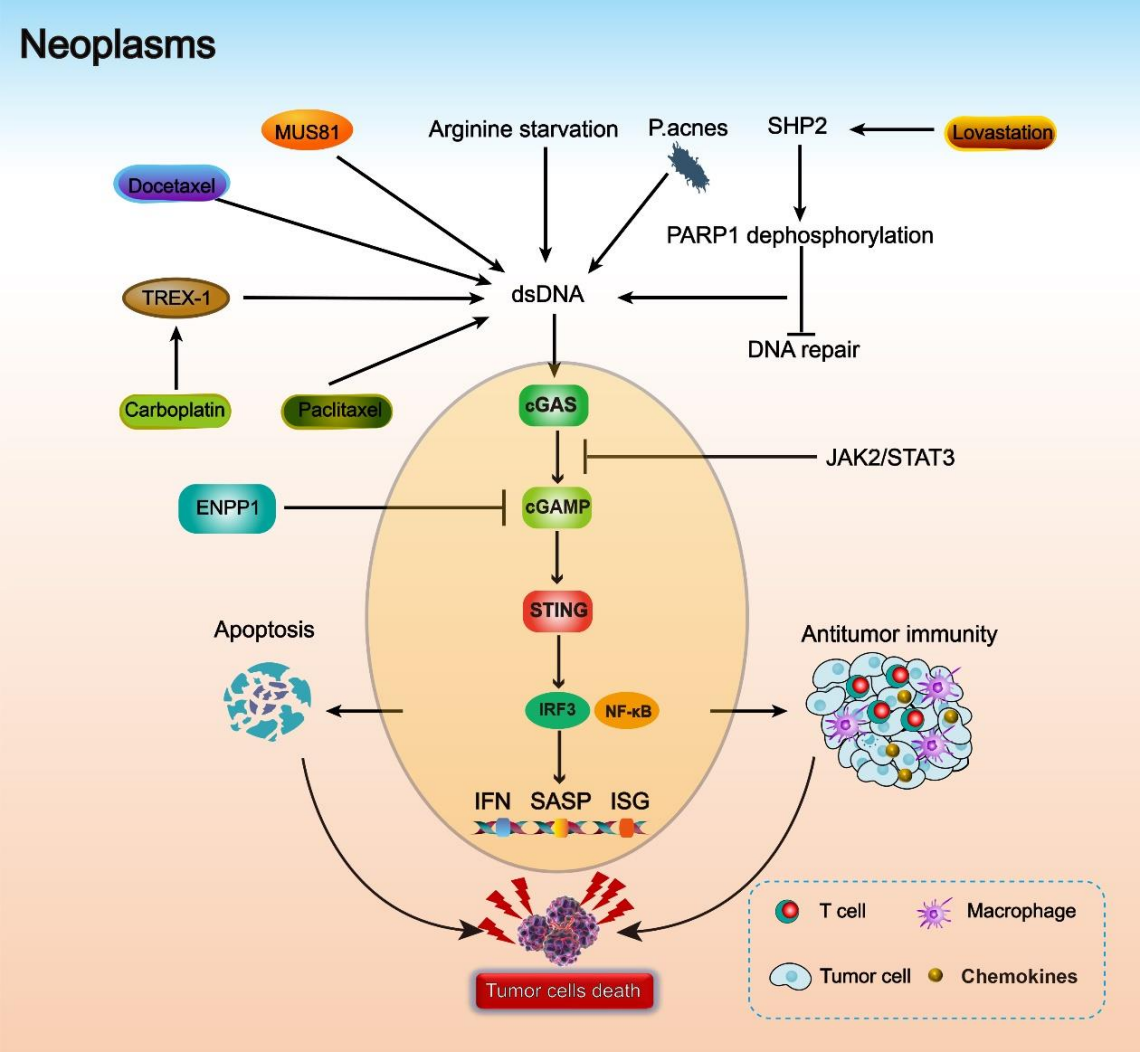
**The cGAS-STING pathway in neoplasms**. MUS81, docetaxel, paclitaxel, arginine starvation, and P. acnes contribute to the increase in dsDNA in the cytoplasm. Carboplatin upregulates the expression of TREX1, which increases cytosolic dsDNA. SHP2 phosphorylates PARP1 and inhibits DNA repair, resulting in the accumulation of dsDNA. Cytosolic dsDNA is recognized by cGAS, and downstream signaling pathways are activated. The activated cGAS-STING pathway induces apoptosis and antitumor immunity, and these effects together lead to tumor cell death. JAK2/STAT3 and ENPP1 can inhibit the cGAS-STING pathway.

Inflammation induced by the cGAS-STING signaling pathway is an essential component of innate immunity that can defend against microbial infections. However, aberrant, and chronic inflammatory stimulation is responsible for some autoimmune diseases, including lethal anemia, polyarthritis, and systemic lupus erythematosus. Recent studies indicated that inflammation plays a crucial role in all phases of tumorigenesis, including onset, progression, malignant invasion, metastasis, morbidity, and mortality [94-96]. The downstream effector TBK1 of the activated cGAS-STING signaling pathway has been recognized as a critical mediator of persistent inflammatory responses that contribute to carcinogenesis (Fig. 4) [97, 98].

### Colorectal cancer (CRC)

CRC is the fourth leading cause of cancer-related deaths worldwide, accounting for approximately 10% of all annually diagnosed cancers and 9.2% of global deaths. Advanced age is a high-risk factor of CRC [99, 100]. Growing evidence suggests that chemotherapy for CRC induces multiple forms of DNA damage, of which DNA double-strand breaks (DSB) are the most severe. If left unrepaired, DSB can lead to mutations that promote an antitumor immune response. Therefore, targeted therapy provides a new strategy for CRC to improve therapeutic efficacy and reduce the recurrence rate. Downregulation of cGAS and STING and decreased transcription of TBK1 and IFN in CRC tissues indicate that the cGAS-STING pathway is inhibited in CRC. The cGAS-STING pathway is essential for the antitumor immunity of CRC, the disruption of which may be a critical mechanism for immune escape of tumor cells [101, 102].

Exogenous cGAMP injection into tumors can provoke the cGAS-STING pathway and promote the expression of IFN, initiating antitumor immunity [103]. Higher STING expression in patients with CRC is associated with longer overall and recurrence-free survival[104]. Zhu et al. generated a colitis-associated CRC mouse model and found that STING-deficient mice exhibited increased colonic inflammation, hyperplasia, tumor burden, and severe intestinal pathology, suggesting that STING plays an essential role in suppressing colorectal tumorigenesis[105]. Poly ADP-ribose polymerase 1 (PARP1) is dephosphorylated by src homology-2 domain-containing protein tyrosine phosphatase-2 (SHP2) when DNA is damaged. This compromises DNA repair and contributes to the accumulation of cytosolic dsDNA, resulting in activation of the cGAS-STING signaling pathway. The agonist lovastatin can enhance the activity of SHP2 and promote the chemotherapeutic effect in colon cancer by activating the cGAS-STING signaling pathway, which could be a better alternative for colon cancer treatment [106]. The cGAS-STING signaling axis may be a potential therapeutic target for CRC [107].

### Melanoma

Melanoma is a potentially fatal malignancy with an increasing incidence and mortality; it usually occurs in young and middle-aged people, and the incidence increases linearly from the age of 25 to 50 years [108-110].

Takashima et al. established a melanoma mouse model by injecting B16D8 melanoma cells into mice, which were sensitive to the cytotoxicity of natural killer (NK) cells. STING-knockout mice and STING-disrupted B16D8 cells were used to probe the critical role of STING in NK cell sensitivity in a melanoma mouse model. The proliferation and survival of STING-disrupted B16D8 cells were unaffected, and the expression of IFNβ mRNA did not increase when stimulated with cGAMP. The growth of STING-disrupted B16D8 cells was much faster than that of normal B16D8 cells in STING knockout mice, indicating that STING is involved in natural regulation and inhibits tumor proliferation[111].

As a primary epigenetic regulator, protein arginine methyltransferase 5 (PRMT5) is involved in various cell growth processes and has been reported to be associated with the development and progression of melanoma. PRMT5 methylation of IFN gamma inducible protein 16 (IFI16) and its mouse homolog IFI204, which plays a vital role in the cGAS-STING pathway, attenuates the production of IFN and chemokines induced by solute DNA, further inhibiting the activity of the cGAS-STING pathway. NLR family CARD domain containing 5 (NLRC5) is a member of a class of genes that regulate MHCI antigen presentation. The elevated NLRC5 expression enhanced MHCI cell surface expression and inhibited melanoma growth more effectively. Furthermore, upregulation of IFI16, IFI204, and NLRC5 could limit melanoma growth in mice and improve the survival of patients with melanoma. PRMT5 function is negatively associated with antitumor immunity. PRMT5 methylates IFI16/IFI204 and inhibits NLRC5 transcription, suppresses inflammation and antigen presentation, and promotes melanoma growth. PRMT5 inhibition antagonizes melanoma growth in immunocompetent mice [112]. TREX1 is essential for DNA replication and repair, but its expression is downregulated in human melanoma cell lines. Carboplatin activates the cGAS-STING pathway by upregulating TREX1 expression to inhibit proliferation and induce apoptosis in melanoma cells. Targeting the TREX1/cGAS-STING signaling axis could be a potential therapy for human melanoma [113].

### Breast tumors

Breast tumors are the most common female malignancy worldwide, accounting for 30% of all female cancers in 2021 [114]. Parkes et al. reported a molecular subtype of breast cancer with a defective DNA damage response (DDR), which demonstrated increased DNA in the cytoplasm and constitutive activation of the cGAS-STING-TBK1-IRF3 pathway [115]. The activated cGAS-STING axis promoted the proliferation of triple-negative breast cancer (TNBC) cells [116].

Chromosomal instability (CIN), a tumor hallmark, is caused by persistent errors in chromosomal segregation during mitosis. It is considered a major driver of metastasis in tumors, such as breast tumors. CIN induced by continuous errors in chromosome segregation contributes to the generation of micronuclei, which are one of the main sources of cytosolic DNA after rupture. Chronic stimulation of cGAS-STING and its downstream effector NF-κB due to CIN is responsible for breast tumor metastasis [117]. Ectonucleotide pyrophosphatase/ phosphodiesterase 1 (ENPP1), which selectively degrades extracellular cGAMP and interferes with immune cell infiltration, is upregulated in TNBC cells with CIN, contributing to resistance to immunotherapy and tumor metastasis [118]. ENPP1 inhibitors may restore innate immune signaling and potentiate antitumor immune responses [119]. Metastatic breast tumor cells in the brain can transfer the second messenger cGAMP to adjacent astrocytes as paracrine signals, which activate STING and mediate the generation of inflammatory cytokines to promote metastatic tumor growth and chemoresistance [120]. Hong et al. found that drug-induced CIN TNBC cells could trigger IL-6 and signal transducer and activator of transcription 3 (STAT3)-mediated signaling to prevent tumor cell death, depending on the activation of the cGAS-STING pathway. Tocilizumab targets the IL-6 receptor and disturbs IL-6-STAT3 signaling, inhibiting the proliferation of TNBC cells with CIN. It has been reported that the application of immune checkpoint inhibitors, such as paclitaxel, in patients with TNBC could induce CIN and the production of micronuclei, which consequently provoked the cGAS-STING pathway. The combined use of tocilizumab with drugs mediating CIN could be a promising therapeutic target for activating IL-6 signaling in cancers, such as TNBC [121, 122].

### Prostate cancer (PCa)

PCa is the second most frequently diagnosed malignancy and the fifth leading cause of cancer-related deaths in men[123]. PCa is recognized as a heterogeneous age-related disorder; the risk of PCa increases significantly with age, and over 85% of newly diagnosed cases are older than 60 years [2, 124]. Recent bioinformatics research has also demonstrated that senescence-related gene prognostic indices can predict PCa prognosis [125].

Cytosolic dsDNA has been detected in various human PCa cells, and dsDNA continually increases after bromodeoxyuridine labeling, demonstrating that genomic DNA is the source of cytosolic DNA in PCa cells[126]. However, STING signaling is defective in human DU145 PCa cells due to the Janus kinase 2 (JAK2)/STAT3 pathway, and STING agonists fail to provoke the cGAS-STING pathway and promote the production of type I IFN. Blocking the IL-6 or JAK2/STAT3 pathway can restore the responsiveness of DU145 cells to STING agonists [127]. DNA structure-specific endonuclease ultraviolet-sensitive 81 (MUS81) is associated with the amount of DNA in the cytoplasm of both human and mouse PCa cells. MUS81 induces the expression of type I IFNs and chemokines, promotes the immune responses of T cells, and enhances phagocytosis of PCa cells in a STING-dependent manner [126]. *Propionibacterium acnes* (*P. acnes*) is the most common microorganism in normal prostate tissues and is abundant in PCa tissues. It can activate the cGAS-STING pathway and induce the expression of IFN, which subsequently promotes PCa growth [128, 129].

Arginine synthesis deficiency, a common characteristic of cancers, causes depletion of α-ketoglutarate and epigenetic silencing of metabolic genes related to oxidative phosphorylation and DNA repair via inactivation of histone demethylases. Cancer cells with arginine deficiency exhibit mitochondrial dysfunction, transcriptional reprogramming, and eventual cell death, resulting in chromatin leakage and DNA damage. This leads to cytosolic DNA accumulation and activation of the cGAS-STING pathway. Moreover, the resulting cGAS-STING activation may further enhance its killing effects. Consequently, PCa growth was inhibited in response to the upregulation of type I IFN and recruitment of immune cells. Arginine starvation can be an effective therapy for PCa by activating the cGAS-STING pathway [130]. Ma et al. combined chemotherapy with checkpoint blockade immunotherapy in PCa to investigate the therapeutic effect and found that docetaxel therapy promoted the expression of IFN, and T cell infiltration mediated by the cGAS-STING pathway in PCa patient samples. Correspondingly, docetaxel therapy enhanced intratumor-infiltrating T cells in a PCa xenograft mouse model and promoted the expression of programmed cell death-1 (PD1) and programmed cell death-ligand 1 (PD-L1), potentiating the anti-PD1 blockade therapeutic effect in PCa xenograft mice. Furthermore, prostate-specific antigen progression-free survival of the combination of docetaxel and anti-PD1 blockade was much better than that of anti-PD1 blockade alone in a metastatic castration-resistant prostate cancer (CRPC) cohort [131].

## Conclusions and perspectives

During human life, genomic stability is challenged by various endogenous and environmental factors. Many mechanisms, collectively named DDR, maintain genomic stability, by recognizing and repairing DNA damage. However, genomic damage still accumulates when genomic damage is excessive and DDR is deficient or disrupted, leading to genomic instability [132]. Genomic instability is a hallmark of aging and a leading cause of age-related diseases, which are closely related to the aberrant presence of cytosolic DNA and activation of the cGAS-STING pathway [3, 133].

Inflammation induced by the cGAS-STING signaling pathway is important for the onset of many age-related diseases. However, the cGAS-STING pathway plays different roles in different age-related diseases (Table 1). Its aberrant activation can lead to pathological cell proliferation, cellular dysfunction, and death, ultimately resulting in a detrimental disease state. Activated cGAS-STING pathway-mediated inflammation increases immune cell infiltration, promotes antitumor immunity, and reduces the risk of tumor metastasis in some cancers. Downregulation or deletion of the cGAS-STING pathway may be an important cause of tumorigenesis and progression. In contrast, the cGAS-STING pathway can also promote tumor proliferation and metastasis [117, 134].

In addition to malignant age-related diseases, inflammation also plays an important role in the development and progression of benign age-related diseases such as benign prostatic hyperplasia (BPH), which mainly develops in the transition zone and enlarges with age. BPH is a hyperplastic process with an increased number of epithelial and stromal cells, but the stromal-to-epithelial ratios in BPH samples are significantly heterogeneous [135]. SASP plays a crucial role in tumorigenesis and age-related diseases. Recent studies have suggested SASP can be triggered by cGAS-STING signaling pathway via accumulation of cytoplasmic DNA in senescent cells. Given the critical role of the cGAS-STING pathway in inflammation and aging, it may also be involved in the development and progression of BPH. Choi et al. used senescent biomarker senescent-associated beta-galactosidase to detect BPH specimens and demonstrated that senescence was only presented in epithelial cells, but not in stromal cells [136]. Although senescent epithelial cells cannot proliferate anymore in BPH, we propose that senescent epithelial cells may secrete SASP via the cGAS-STING pathway to promote the proliferation of stromal cells, which could be a potential mechanism by which aging results in BPH [137, 138]. However, given that there are no studies have been reported on the cGAS-STING pathway and BPH, further studies are needed to confirm the hypothesis.

**Table 1:** List of age-related diseases and supporting evidence associated with the cGAS-STING pathway.

Age-related disease		cGAS-STING activity	Pathogenesis	Human sample	Animal model	Therapy	Therapeutic effect	Ref
**Cardiovascular diseases**	Cardiac hypertrophy	In human: upregulated STING and type I IFNs In animal: upregulated STING and type I IFNs	-	DCM and HCM patients	Cardiac hypertrophy mice induced by AB and Ang II	STING knockout	Alleviating inflammation, fibrosis, and attenuating cardiac hypertrophy	[31]
		In animal: upregulated cGAS, cGAMP, TBK1, IRF3, type I IFN, TNF-α, IL-1β and IL-18	Free fatty acids	-	DCM mice induced by streptozotocin and HFD	cGAS and STING knockdown and inhibitor (Ru.521 and C176-1)	Alleviating myocardial pyroptosis, inflammation, and attenuating cardiac hypertrophy	[32]
		In animal: upregulated cGAS, STING, TNFαand IL-1β	Akt2-AMPKα2 double ablation	-	Akt2-/- and AMPKα2-/- mice; HFD-induced cardiac hypertrophy mice	cGAS and STING inhibitors (PF-06928215 and Astin C)	Ameliorating HFD-induced contractile dysfunction	[33]
	Atherosclerosis	In human: upregulated of NF-κB, IL-6 and TNF-α In animal: downregulated IL-6 and TNF-α after TDP43 knockout	TDP43 mis-localization	CAD patients peripheral blood sample	TDP43-/- mice	NF-κB inhibitors (PDTC and SC75741)	Alleviating AS in TDP43-/- mice	[40]
	Myocardial infarction	In animal: downregulated type I IFN in IRF3 knockout mice, upregulated ISGs in WT mice	Dead myocardial cells	-	cGAS and STING deficient mice	Genetic disrupton or antibody blockade of IFNAR1	Improving heart function and survival	[44]
		In human: upregulated cGAS and CXCL10 In animal: upregulated STING, CXCL10,IFIT1,IFIT3, IRF7 and CD14	Dead myocardial cells	Failing and nonfailing hearts tissues	LAD coronary artery ligation-induced MI mice; cGAS-null mice	cGAS knockout	Improving myocardial repair after infarction	[45]
	Aortic aneurysm and dissection	In human: upregulated STING, TBK1 and IRF3 In animal: upregulated STING	DNA from damaged SMCs	AAD patient aortic tissues	HFD and Ang II-induced AAD model; Sting deficient mice	TBK1 Inhibitor (amlexanox)	Partially preventing AAD development	[51]
**Neurological disorders**	Alzheimer’s disease	In human: upregulated type I IFN, TNFα, IL-1β, and IL-6 In animal: upregulated type I IFN	-	AD patient prefrontal cortex	AD mouse model; IFNAR1-/- mice	IFNAR1 ablation	Protection against Aβ-induced toxicity	[57] [59]
	Huntington’s disease	In human: upregulated cGAS, STING and IRF3 In animal: upregulated cGAS, STING, IRF3, type I IFN, IL-1β, IL-6 and IL-18	Melatonin decrease	HD patient striatum samples	HD mice model	cGAS inhibitor (Ru.521); melatonin	Inhibition of cGAS-STING pathway	[67]
		In animal: upregulated cGAS, STING and IRF3	-	-	TOLLIP-/- mice	TOLLIP knockout	Impairing STING signaling and ameliorating HD	[70]
	Parkinson’s disease	In LRRK2 KO macrophages: cGAS, STING, IRF3, IFN and ISG	LRRK2 mutation	-	LRRK2 knockout mice	reducing DRP1 and antioxidants	Alleviating mitochondrial damage and decreasing type I IFN	[78]
		In human: upregulated IL-6, IL-1β, CCL2, and CCL4 In animal: upregulated cGAMP, IL-6, IFNβ1, IL-12, IL-13, CXCL1, CCL2, and CCL4	Parkin and PINK1 mutations	-	Parkin-/-, PINK1-/- and STING-/- mice	Blocking IFNAR1 or STING knockout	Alleviating inflammation and PD	[80]
	Aicardi-Goutières syndrome	In animal: upregulated cGAMP and IFN	TREX1 mutation	-	TREX1 deficient mice	IFNAR1 knockout	The mortality and pathology significantly improved	[86-87]
	Ataxia-telangiectasia	In brain organoid: downregulated cGAS, STING and SASP (IL-6, IL-8, IL1α and CCL20) via RNA interference	ATM mutation	A-T patient nasal biopsies	-	cGAS and STING inhibitors (aspirin and H-151)	Reduction of senescence phenotypes and inflammation	[92]
		In animal: upregulated STING, TBK1, NF-κB, IFNβ, IL-1β	ATM mutation	-	ATM knockout mice	Steroids (betamethasone)	Reducing the loss of motor neurons	[93]
**Neoplasms**	Colorectal cancer	In cells: downregulated cGAS, STING, IRF3 and IFN	-	-	STING knockout mice	Viral oncolytic therapy	Tumor cells growth reduction	[101]
		In cells: upregulated STING and IFN after cGAMP stimulation	-	-	shSHP2 mice	SHP2 agonist (lovastatin)	Delayed DNA damage repair and enhanced antitumor immunity	[106]
	Melanoma	In cell: upregulated cGAS, STING, TBK1, IRF3 and TNF after TREX1 overexpression	TREX1 decrease	Human sinonasal mucosal melanoma	-	Carboplatin	Inducing apoptosis and inhibiting proliferation	[113]
	Breast tumors	In human: upregulated cGAS, STING, TBK1, IRF3	CIN	Human breast cancer samples	-	Tocilizumab	Inducing tumor cells death	[121]
	Prostate cancer	In cell: upregulated IRF3 and type I IFN	Nuclear MUS81 decrease	Human PCa samples	-	-	-	[126]
		In cells: upregulated cGAS, STING, TBK1 and type I IFN	Arginine	-	Arginine-restriction xenograft mouse model	Arginine starvation	Increased immune cells infiltration and PCa growth inhibition	[130]
		In human: upregulated cGAS, STING and IFN	-	PCa patient tumor samples	CRPC mice	Docetaxel	Immune microenvironment remodeling and enhancing immunotherapy	[131]

For each age-related disease described in the text, the evidence for causality or correlation with the cGAS-STING pathway, human sample(s), animal model(s), therapy targeting this pathway and its effect are listed. cGAS, cyclic guanosine monophosphate (GMP)- adenosine monophosphate (AMP) synthase; cGAMP, cyclic guanosine monophosphate-adenosine monophosphate; STING, stimulator of interferon genes; IFN, interferon; TBK1, TANK-binding kinase 1; IRF3, interferon regulatory factor 3; IL, interleukin; TNF, tumor necrosis factor; DCM, diabetic cardiomyopathy; HCM, hypertrophic cardiomyopathy; AB, aortic banding; Ang II, angiotensin II; NLRP3, nucleotide-binding oligomerization domain-like receptor pyrin domain containing 3; Akt2, protein kinase B 2; AMPK, AMP-dependent protein kinase; TDP43, Transactive response DNA-binding protein ~43 kDa; CAD, coronary artery disease; ISG, interferon stimulated gene; CXCL10, chemokine (C-X-C motif) Ligand 10; IFIT, interferon-induced protein with tetratricopeptide repeat; CD14, cluster of differentiation 14; AAD, aortic aneurysm and dissection; HFD, high-fat diet; TOLLIP, toll-interacting protein; MI, myocardial infarction; SMCs, smooth muscle cells; MMP-9, matrix metalloproteinase (MMP)-9; AD, Alzheimer’s disease; IFNAR1, type I interferon-α receptor 1; HD,Huntington’s disease; LRRK2, leucine-rich repeat kinase 2; DRP1, dynamin-related protein 1; SHP2, Src homology-2 domain-containing protein tyrosine phosphatase-2; TREX1, three-prime repair exonuclease 1; CIN, chromosomal instability; MUS81, DNA structure-specific endonuclease ultraviolet-sensitive 81; CRPC, castration-resistant prostate cancer.

Treatment with inhibitors or agonists targeting the cGAS-STING pathway in age-related diseases should be prudent depending on the function of this pathway in these diseases. Some drugs targeting the cGAS-STING pathway used for the treatment of diseases are listed in Table 2 [87, 103, 139-159]. However, as most of these drugs are still in the experimental/clinical trial stage, they cannot be used in humans to address aging and age-related diseases [160]. Further studies are needed to identify ideal inhibitors or agonists targeting the cGAS-STING pathway that can be used in humans. These agents may provide new insights into and therapeutic alternatives for age-related diseases.

**Table 2:** The inhibitors and agonists of the cGAS-STING pathway.

Target	Agents	Function or property	Species specificity	Advantage	Disadvantage	Application model	Treatment effect	Ref
**cGAS inhibitors**	AMDs	Blocking dsDNA/cGAS binding	Human and mouse cGAS	Widely used with a strong safety profile	-	Trex1-/- mouse model	Effective for the treatment of AGS mice	[87]
	EGCG	Disrupting cGAS activation	Human and mouse cGAS	Specifically inhibit inflammation caused by cGAS activation	Effect of EGCG on cGAS activity is clearly dependent on G3BP1	AGS patient cells and AGS mouse model	Effective in treating cGAS-mediated autoinflammation	[139]
	Aspirin	Inhibiting cGAS by acetylation	Human and mouse cGAS	Widely used with a strong safety profile	-	AGS patient cells and AGS mouse model	Effective in treating AGS and potentially other DNA-mediated autoimmune diseases	[140]
	RU.521	Occupying the catalytic site of cGAS	Mouse cGAS	Selective inhibition of cGAS dsDNA-dependent enzymatic activity in vitro and in cells	Poor inhibitor of recombinant human cGAS	AGS mouse model	Suppressing type I IFN expression	[141]
	G140, G150	Inhibiting cGAS activity	Human cGAS	Specific and potent small-molecule inhibitors for human cGAS	The utility for therapeutic treatment of cGAS-related human diseases is uncertain	Human monocytic THP1 cells and mouse macrophage RAW 264.7 cells	Inhibiting dsDNA-induced cGAS activity	[142]
	PAH	Inhibiting cGAS activity	Human and mouse cGAS	No side effects and toxicity with biological safety in vivo	Inhibitory mechanisms are uncertain	AGS mouse model	Ameliorating self-DNA-induced autoinflammatory responses	[143]
	Compound 25	Inhibiting cGAS activity	Human and mouse cGAS	Superior in vivo anti-inflammatory effects	Structural optimization is needed to achieve higher potency	Lipopolysaccharide-induced mouse model	Ihibiting the dsDNA-induced phosphorylation of STING/TBK1/IRF3 signaling and the mRNA expression of ISGs	[144]
	A151	Competing with DNA	Mouse cGAS	Into the damaged brain parenchyma from the blood	-	Ischemic mouse brains model	Protecting against brain damage and improving neurodeficits	[145]
**STING inhibitors**	Compound 18	Competing with cGAMP	Human STING	Good oral exposure and slow binding kinetics	-	Human monocytic THP1 cells	Inhibiting cGAMP-induced IFNβ production	[146]
	Astin C	Blocking IRF3 recruitment onto the STING	Human and mouse STING	Well-tolerated compound with minimal cytotoxic side effects	The in vivo effectiveness is uncertain	Trex1-/- BMDMs cells and Trex1-/- mouse model	Inhibiting the expression of type I IFN and pro-inflammatory cytokines and alleviating the autoinflammatory responses	[147]
	NO2-FA	Inhibiting STING palmitoylation	Human and mouse STING	Natural antiinflammatory mediators	Not yet used in clinical practice	Fibroblasts from SAVI patients	Inhibiting production of type I IFN	[148]
	C-178, C-176	Inhibiting STING palmitoylation	Mouse STING	In vivo inhibitory capacity is not limited by the short serum half-life	Low affinity to human STING	Trex1-/- mouse model	Amelioration of various signs of systemic inflammation	[149]
	C-170, C-171, H-151	Blocking palmitoylation-induced clustering of STING	Human and mouse STING	Highly potent and selective small-molecule inhibitor	-	Trex1-/- mouse model	Reducing systemic cytokine responses	[149]
	SN-011	Blocking CDN binding and STING activation	Human and mouse STING	Potent and selective inhibitor with high affinity and specificity	-	Trex1-/- mouse model	Ameliorating autoimmune pathology and preventing death	[150]
**STING agonists**	DMXAA	Non-CDNs	Mouse STING	Potent and specific therapeutic effect on mice	Lacking the ability to activate human STING	Mouse xenotransplantation models	Induction of cytokines and disrupting tumor vascularization	[151]
	CMA	Non-CDNs	Mouse STING	Potent and specific therapeutic effect on mice	Lacking the ability to activate human STING	Mouse macrophages	Inducing IRF3 phosphorylation and Ifnb mRNA translation	[152]
	c(di-GMP)	CDNs	Mouse STING	Mobilizing abscopal immunity when combined with checkpoint modulation	Low affinity to human STING	Bilaterally-implanted TRAMP-C2 tumors mouse model	Mediating regression of injected tumors	[153]
	3'3'-cGAMP	CDNs	Mouse STING	More efficient than DMXAA in activating STING	-	Chronic lymphocytic leukemia and multiple myeloma mouse model	Inducing apoptosis and tumor regression	[154]
	ML RR-S2 CDA	CDNs	Human and mouse STING	Activating all human and mouse STING alleles	Intratumor injection is necessary to achieve maximal therapeutic effect	4T-1 colon or CT26 mammary carcinomas mouse model	Inducing tumor regression and resistant to the same tumor cell line	[103]
	ABZI	Non-CDNs	Human and mouse STING	Intravenous administration can induce adaptive CD8 T cell response in vivo	-	Syngeneic colon tumours mouse model	Strong anti-tumour activity with complete and lasting regression of tumours	[155]
	STING-LNP	Non-CDNs	Mouse STING	Efficiently delivered the STING agonist to the cytoplasm	-	B16-F10-luc2 lung metastatic mouse model	Increasing the expression of CD3, CD4, PD-1 and IFN in lung metastases	[156]
	MSA-2	Non-CDNs	Human and mouse STING	Favorable activity and tolerability profiles	-	MC38 syngeneic tumors mouse model	Inducing tumor regressions	[157]
	PC7A	Non-CDNs	Human and mouse STING	Polyvalent STING agonist with prolonged cytokine expression	-	MC38 and TC-1 tumour models	Notably extended the survival	[158]
	SR-717	Non-CDNs	Human and mouse STING	Substantial efficacy without considerable toxicity	-	B16.F10 melanomas mouse model	Reduction in tumor growth and the increased survival	[159]

cGAS, cyclic guanosine monophosphate (GMP)- adenosine monophosphate (AMP) synthase; cGAMP, cyclic guanosine monophosphate-adenosine monophosphate; STING, stimulator of interferon genes; IFN, interferon; TBK1, TANK-binding kinase 1; IRF3, interferon regulatory factor 3; ISG, interferon stimulated gene; AMDs: antimalarial drugs; EGCG: epigallocatechin gallate; PAH: perillaldehyde; BMDMs: bone marrow-derived macrophages; SAVI: STING-associated vasculopathy with onset in infancy; CDN: cyclic dinucleotide; ABZI: amidobenzimidazole.

## References

[1] McHugh D, Gil J (2018). Senescence and aging: Causes, consequences, and therapeutic avenues. J Cell Biol, 217:65-77. 29114066 10.1083/jcb.201708092 PMC5748990

[2] Chang AY, Skirbekk VF, Tyrovolas S, Kassebaum NJ, Dieleman JL (2019). Measuring population ageing: an analysis of the Global Burden of Disease Study 2017. Lancet Public Health, 4: e159-e167. 30851869 10.1016/S2468-2667(19)30019-2 PMC6472541

[3] López-Otín C, Blasco MA, Partridge L, Serrano M, Kroemer G (2013). The hallmarks of aging. Cell, 153:1194-1217. 23746838 10.1016/j.cell.2013.05.039 PMC3836174

[4] Hoeijmakers JH (2009). DNA damage, aging, and cancer. N Engl J Med, 361:1475-1485. 19812404 10.1056/NEJMra0804615

[5] Moskalev AA, Shaposhnikov MV, Plyusnina EN, Zhavoronkov A, Budovsky A, Yanai H, et al. (2013). The role of DNA damage and repair in aging through the prism of Koch-like criteria. Ageing Res Rev, 12:661-684. 22353384 10.1016/j.arr.2012.02.001

[6] Lord CJ, Ashworth A (2012). The DNA damage response and cancer therapy. Nature, 481:287-294. 22258607 10.1038/nature10760

[7] Blackburn EH, Greider CW, Szostak JW (2006). Telomeres and telomerase: the path from maize, Tetrahymena and yeast to human cancer and aging. Nat Med, 12:1133-1138. 17024208 10.1038/nm1006-1133

[8] Kazak L, Reyes A, Holt IJ (2012). Minimizing the damage: repair pathways keep mitochondrial DNA intact. Nat Rev Mol Cell Biol, 13:659-671. 22992591 10.1038/nrm3439

[9] Green DR, Galluzzi L, Kroemer G (2011). Mitochondria and the autophagy-inflammation-cell death axis in organismal aging. Science, 333:1109-1112. 21868666 10.1126/science.1201940 PMC3405151

[10] Miller KN, Victorelli SG, Salmonowicz H, Dasgupta N, Liu T, Passos JF, et al. (2021). Cytoplasmic DNA: sources, sensing, and role in aging and disease. Cell, 184:5506-5526. 34715021 10.1016/j.cell.2021.09.034 PMC8627867

[11] Akbari M, Shanley DP, Bohr VA, Rasmussen LJ (2021). Cytosolic Self-DNA-A Potential Source of Chronic Inflammation in Aging. Cells, 10. 34944052 10.3390/cells10123544 PMC8700131

[12] Sun L, Wu J, Du F, Chen X, Chen ZJ (2013). Cyclic GMP-AMP synthase is a cytosolic DNA sensor that activates the type I interferon pathway. Science, 339:786-791. 23258413 10.1126/science.1232458 PMC3863629

[13] Decout A, Katz JD, Venkatraman S, Ablasser A (2021). The cGAS-STING pathway as a therapeutic target in inflammatory diseases. Nat Rev Immunol, 21:548-569. 33833439 10.1038/s41577-021-00524-z PMC8029610

[14] Wu J, Sun L, Chen X, Du F, Shi H, Chen C, et al. (2013). Cyclic GMP-AMP is an endogenous second messenger in innate immune signaling by cytosolic DNA. Science, 339:826-830. 23258412 10.1126/science.1229963 PMC3855410

[15] Ishikawa H, Barber GN (2008). STING is an endoplasmic reticulum adaptor that facilitates innate immune signalling. Nature, 455:674-678. 18724357 10.1038/nature07317 PMC2804933

[16] Fang R, Jiang Q, Guan Y, Gao P, Zhang R, Zhao Z, et al. (2021). Golgi apparatus-synthesized sulfated glycosaminoglycans mediate polymerization and activation of the cGAMP sensor STING. Immunity, 54:962-975.e968. 33857420 10.1016/j.immuni.2021.03.011

[17] Zhang X, Bai XC, Chen ZJ (2020). Structures and Mechanisms in the cGAS-STING Innate Immunity Pathway. Immunity, 53:43-53. 32668227 10.1016/j.immuni.2020.05.013

[18] Birch J, Gil J (2020). Senescence and the SASP: many therapeutic avenues. Genes Dev, 34:1565-1576. 33262144 10.1101/gad.343129.120 PMC7706700

[19] Kuilman T, Peeper DS (2009). Senescence-messaging secretome: SMS-ing cellular stress. Nat Rev Cancer, 9:81-94. 19132009 10.1038/nrc2560

[20] Dou Z, Ghosh K, Vizioli MG, Zhu J, Sen P, Wangensteen KJ, et al. (2017). Cytoplasmic chromatin triggers inflammation in senescence and cancer. Nature, 550:402-406. 28976970 10.1038/nature24050 PMC5850938

[21] Glück S, Guey B, Gulen MF, Wolter K, Kang TW, Schmacke NA, et al. (2017). Innate immune sensing of cytosolic chromatin fragments through cGAS promotes senescence. Nat Cell Biol, 19:1061-1070. 28759028 10.1038/ncb3586 PMC5826565

[22] Xue W, Zender L, Miething C, Dickins RA, Hernando E, Krizhanovsky V, et al. (2007). Senescence and tumour clearance is triggered by p53 restoration in murine liver carcinomas. Nature, 445:656-660. 17251933 10.1038/nature05529 PMC4601097

[23] Krtolica A, Parrinello S, Lockett S, Desprez PY, Campisi J (2001). Senescent fibroblasts promote epithelial cell growth and tumorigenesis: a link between cancer and aging. Proc Natl Acad Sci U S A, 98:12072-12077. 11593017 10.1073/pnas.211053698 PMC59769

[24] Coppé JP, Patil CK, Rodier F, Sun Y, Muñoz DP, Goldstein J, et al. (2008). Senescence-associated secretory phenotypes reveal cell-nonautonomous functions of oncogenic RAS and the p53 tumor suppressor. PLoS Biol, 6:2853-2868. 19053174 10.1371/journal.pbio.0060301 PMC2592359

[25] Evans MA, Sano S, Walsh K (2020). Cardiovascular Disease, Aging, and Clonal Hematopoiesis. Annu Rev Pathol, 15:419-438. 31689371 10.1146/annurev-pathmechdis-012419-032544 PMC7104598

[26] Donato AJ, Machin DR, Lesniewski LA (2018). Mechanisms of Dysfunction in the Aging Vasculature and Role in Age-Related Disease. Circ Res, 123:825-848. 30355078 10.1161/CIRCRESAHA.118.312563 PMC6207260

[27] Oduro PK, Zheng X, Wei J, Yang Y, Wang Y, Zhang H, et al. (2022). The cGAS-STING signaling in cardiovascular and metabolic diseases: Future novel target option for pharmacotherapy. Acta Pharm Sin B, 12:50-75. 35127372 10.1016/j.apsb.2021.05.011 PMC8799861

[28] Shimizu I, Minamino T (2016). Physiological and pathological cardiac hypertrophy. J Mol Cell Cardiol, 97:245-262. 27262674 10.1016/j.yjmcc.2016.06.001

[29] Hu D, Cui YX, Wu MY, Li L, Su LN, Lian Z, et al. (2020). Cytosolic DNA sensor cGAS plays an essential pathogenetic role in pressure overload-induced heart failure. Am J Physiol Heart Circ Physiol, 318:H1525-h1537. 32383996 10.1152/ajpheart.00097.2020

[30] Sun H, Wang Y (2014). Interferon regulatory factors in heart: stress response beyond inflammation. Hypertension, 63:663-664. 24396026 10.1161/HYPERTENSIONAHA.113.02795 PMC4046326

[31] Zhang Y, Chen W, Wang Y (2020). STING is an essential regulator of heart inflammation and fibrosis in mice with pathological cardiac hypertrophy via endoplasmic reticulum (ER) stress. Biomed Pharmacother, 125:110022. 32106379 10.1016/j.biopha.2020.110022

[32] Yan M, Li Y, Luo Q, Zeng W, Shao X, Li L, et al. (2022). Mitochondrial damage and activation of the cytosolic DNA sensor cGAS-STING pathway lead to cardiac pyroptosis and hypertrophy in diabetic cardiomyopathy mice. Cell Death Discov, 8:258. 35538059 10.1038/s41420-022-01046-w PMC9091247

[33] Gong Y, Li G, Tao J, Wu NN, Kandadi MR, Bi Y, et al. (2020). Double knockout of Akt2 and AMPK accentuates high fat diet-induced cardiac anomalies through a cGAS-STING-mediated mechanism. Biochim Biophys Acta Mol Basis Dis, 1866:165855. 32512189 10.1016/j.bbadis.2020.165855

[34] Rech L, Abdellatif M, Pöttler M, Stangl V, Mabotuwana N, Hardy S, et al. (2022). Small molecule STING inhibition improves myocardial infarction remodeling. Life Sci, 291:120263. 34971697 10.1016/j.lfs.2021.120263

[35] Han W, Du C, Zhu Y, Ran L, Wang Y, Xiong J, et al. (2022). Targeting Myocardial Mitochondria-STING-Polyamine Axis Prevents Cardiac Hypertrophy in Chronic Kidney Disease. JACC Basic Transl Sci, 7:820-840. 36061341 10.1016/j.jacbts.2022.03.006 PMC9436763

[36] Falk E (2006). Pathogenesis of atherosclerosis. J Am Coll Cardiol, 47:C7-12. 16631513 10.1016/j.jacc.2005.09.068

[37] Tabares-Guevara JH, Villa-Pulgarin JA, Hernandez JC (2021). Atherosclerosis: immunopathogenesis and strategies for immunotherapy. Immunotherapy, 13:1231-1244. 34382409 10.2217/imt-2021-0009

[38] Zhu Y, Xian X, Wang Z, Bi Y, Chen Q, Han X, et al. (2018). Research Progress on the Relationship between Atherosclerosis and Inflammation. Biomolecules, 8. 10.3390/biom8030080 PMC6163673 30142970

[39] Libby P, Ridker PM, Hansson GK (2011). Progress and challenges in translating the biology of atherosclerosis. Nature, 473:317-325. 21593864 10.1038/nature10146

[40] Huangfu N, Wang Y, Xu Z, Zheng W, Tao C, Li Z, et al. (2021). TDP43 Exacerbates Atherosclerosis Progression by Promoting Inflammation and Lipid Uptake of Macrophages. Front Cell Dev Biol, 9:687169. 34291051 10.3389/fcell.2021.687169 PMC8287832

[41] Wan X, Tian J, Hao P, Zhou K, Zhang J, Zhou Y, et al. (2022). cGAS-STING Pathway Performance in the Vulnerable Atherosclerotic Plaque. Aging Dis, 13:1606-1614. 36465175 10.14336/AD.2022.0417 PMC9662268

[42] Bi X, Du C, Wang X, Wang XY, Han W, Wang Y, et al. (2021). Mitochondrial Damage-Induced Innate Immune Activation in Vascular Smooth Muscle Cells Promotes Chronic Kidney Disease-Associated Plaque Vulnerability. Adv Sci (Weinh), 8:2002738. 33717842 10.1002/advs.202002738 PMC7927614

[43] Thygesen K, Alpert JS, White HD (2007). Universal definition of myocardial infarction. J Am Coll Cardiol, 50:2173-2195. 18036459 10.1016/j.jacc.2007.09.011

[44] King KR, Aguirre AD, Ye YX, Sun Y, Roh JD, Ng RP Jr., et al. (2017). IRF3 and type I interferons fuel a fatal response to myocardial infarction. Nat Med, 23:1481-1487. 29106401 10.1038/nm.4428 PMC6477926

[45] Cao DJ, Schiattarella GG, Villalobos E, Jiang N, May HI, Li T, et al. (2018). Cytosolic DNA Sensing Promotes Macrophage Transformation and Governs Myocardial Ischemic Injury. Circulation, 137:2613-2634. 29437120 10.1161/CIRCULATIONAHA.117.031046 PMC5997506

[46] Yan X, Anzai A, Katsumata Y, Matsuhashi T, Ito K, Endo J, et al. (2013). Temporal dynamics of cardiac immune cell accumulation following acute myocardial infarction. J Mol Cell Cardiol, 62:24-35. 23644221 10.1016/j.yjmcc.2013.04.023

[47] Lawrence T, Natoli G (2011). Transcriptional regulation of macrophage polarization: enabling diversity with identity. Nat Rev Immunol, 11:750-761. 22025054 10.1038/nri3088

[48] Skotsimara G, Antonopoulos A, Oikonomou E, Papastamos C, Siasos G, Tousoulis D (2022). Aortic Wall Inflammation in the Pathogenesis, Diagnosis and Treatment of Aortic Aneurysms. Inflammation, 45:965-976. 35076833 10.1007/s10753-022-01626-z

[49] Chen MT, Chung CH, Ke HY, Peng CK, Chien WC, Shen CH (2021). Risk of Aortic Aneurysm and Dissection in Patients with Tuberculosis: A Nationwide Population-Based Cohort Study. Int J Environ Res Public Health, 18. 34769592 10.3390/ijerph182111075 PMC8583242

[50] Guo DC, Papke CL, He R, Milewicz DM (2006). Pathogenesis of thoracic and abdominal aortic aneurysms. Ann N Y Acad Sci, 1085:339-352. 17182954 10.1196/annals.1383.013

[51] Luo W, Wang Y, Zhang L, Ren P, Zhang C, Li Y, et al. (2020). Critical Role of Cytosolic DNA and Its Sensing Adaptor STING in Aortic Degeneration, Dissection, and Rupture. Circulation, 141:42-66. 31887080 10.1161/CIRCULATIONAHA.119.041460 PMC6939474

[52] Paul BD, Snyder SH, Bohr VA (2021). Signaling by cGAS-STING in Neurodegeneration, Neuroinflammation, and Aging. Trends Neurosci, 44:83-96. 33187730 10.1016/j.tins.2020.10.008 PMC8662531

[53] Lane CA, Hardy J, Schott JM (2018). Alzheimer's disease. Eur J Neurol, 25:59-70. 28872215 10.1111/ene.13439

[54] Scheltens P, De Strooper B, Kivipelto M, Holstege H, Chételat G, Teunissen CE, et al. (2021). Alzheimer's disease. Lancet, 397:1577-1590. 33667416 10.1016/S0140-6736(20)32205-4 PMC8354300

[55] Taylor JM, Moore Z, Minter MR, Crack PJ (2018). Type-I interferon pathway in neuroinflammation and neurodegeneration: focus on Alzheimer's disease. J Neural Transm (Vienna), 125:797-807. 28676934 10.1007/s00702-017-1745-4

[56] Baruch K, Deczkowska A, David E, Castellano JM, Miller O, Kertser A, et al. (2014). Aging. Aging-induced type I interferon response at the choroid plexus negatively affects brain function. Science, 346:89-93. 25147279 10.1126/science.1252945 PMC4869326

[57] Mesquita SD, Ferreira AC, Gao F, Coppola G, Geschwind DH, Sousa JC, et al. (2015). The choroid plexus transcriptome reveals changes in type I and II interferon responses in a mouse model of Alzheimer's disease. Brain Behav Immun, 49:280-292. 26092102 10.1016/j.bbi.2015.06.008

[58] Minter MR, Moore Z, Zhang M, Brody KM, Jones NC, Shultz SR, et al. (2016). Deletion of the type-1 interferon receptor in APPSWE/PS1ΔE9 mice preserves cognitive function and alters glial phenotype. Acta Neuropathol Commun, 4:72. 27400725 10.1186/s40478-016-0341-4 PMC4940712

[59] Taylor JM, Minter MR, Newman AG, Zhang M, Adlard PA, Crack PJ (2014). Type-1 interferon signaling mediates neuro-inflammatory events in models of Alzheimer's disease. Neurobiol Aging, 35:1012-1023. 24262201 10.1016/j.neurobiolaging.2013.10.089

[60] Minter MR, Main BS, Brody KM, Zhang M, Taylor JM, Crack PJ (2015). Soluble amyloid triggers a myeloid differentiation factor 88 and interferon regulatory factor 7 dependent neuronal type-1 interferon response in vitro. J Neuroinflammation, 12:71. 25879763 10.1186/s12974-015-0263-2 PMC4407532

[61] Chen K, Lai C, Su Y, Bao WD, Yang LN, Xu PP, et al. (2022). cGAS-STING-mediated IFN-I Response in Host Defense and Neuroinflammatory Diseases. Curr Neuropharmacol, 20:362-371. 34561985 10.2174/1570159X19666210924110144 PMC9413793

[62] McColgan P, Tabrizi SJ (2018). Huntington's disease: a clinical review. Eur J Neurol, 25:24-34. 28817209 10.1111/ene.13413

[63] Walker FO (2007). Huntington's disease. Lancet, 369:218-228. 17240289 10.1016/S0140-6736(07)60111-1

[64] CAG Repeat Not Polyglutamine Length Determines Timing of Huntington's Disease Onset (2019). Cell, 178:887-900.e814. 31398342 10.1016/j.cell.2019.06.036 PMC6700281

[65] Correia K, Harold D, Kim KH, Holmans P, Jones L, Orth M, et al. (2015). The Genetic Modifiers of Motor OnsetAge (GeM MOA) Website: Genome-wide Association Analysis for Genetic Modifiers of Huntington's Disease. J Huntingtons Dis, 4:279-284. 26444025 10.3233/JHD-150169 PMC4753529

[66] Yablonska S, Ganesan V, Ferrando LM, Kim J, Pyzel A, Baranova OV, et al. (2019). Mutant huntingtin disrupts mitochondrial proteostasis by interacting with TIM23. Proc Natl Acad Sci U S A, 116:16593-16602. 31346086 10.1073/pnas.1904101116 PMC6697818

[67] Jauhari A, Baranov SV, Suofu Y, Kim J, Singh T, Yablonska S, et al. (2020). Melatonin inhibits cytosolic mitochondrial DNA-induced neuroinflammatory signaling in accelerated aging and neurodegeneration. J Clin Invest, 130:3124-3136. 32182222 10.1172/JCI135026 PMC7260019

[68] Acevedo-Torres K, Berríos L, Rosario N, Dufault V, Skatchkov S, Eaton MJ, et al. (2009). Mitochondrial DNA damage is a hallmark of chemically induced and the R6/2 transgenic model of Huntington's disease. DNA Repair (Amst), 8:126-136. 18935984 10.1016/j.dnarep.2008.09.004 PMC3268004

[69] Li M, Feng B, Wang L, Guo S, Zhang P, Gong J, et al. (2015). Tollip is a critical mediator of cerebral ischaemia-reperfusion injury. J Pathol, 237:249-262. 26011492 10.1002/path.4565

[70] Pokatayev V, Yang K, Tu X, Dobbs N, Wu J, Kalb RG, et al. (2020). Homeostatic regulation of STING protein at the resting state by stabilizer TOLLIP. Nat Immunol, 21:158-167. 31932809 10.1038/s41590-019-0569-9 PMC6983345

[71] Samii A, Nutt JG, Ransom BR (2004). Parkinson's disease. Lancet, 363:1783-1793. 15172778 10.1016/S0140-6736(04)16305-8

[72] Tolosa E, Garrido A, Scholz SW, Poewe W (2021). Challenges in the diagnosis of Parkinson's disease. Lancet Neurol, 20:385-397. 33894193 10.1016/S1474-4422(21)00030-2 PMC8185633

[73] Fearnley JM, Lees AJ (1991). Ageing and Parkinson's disease: substantia nigra regional selectivity. Brain, 114(Pt 5):2283-2301. 1933245 10.1093/brain/114.5.2283

[74] Sampson TR, Debelius JW, Thron T, Janssen S, Shastri GG, Ilhan ZE, et al. (2016). Gut Microbiota Regulate Motor Deficits and Neuroinflammation in a Model of Parkinson's Disease. Cell, 167:1469-1480.e1412. 27912057 10.1016/j.cell.2016.11.018 PMC5718049

[75] Gelders G, Baekelandt V, Van der Perren A (2018). Linking Neuroinflammation and Neurodegeneration in Parkinson's Disease. J Immunol Res, 2018:4784268. 29850629 10.1155/2018/4784268 PMC5926497

[76] Singleton AB, Farrer MJ, Bonifati V (2013). The genetics of Parkinson's disease: progress and therapeutic implications. Mov Disord, 28:14-23. 23389780 10.1002/mds.25249 PMC3578399

[77] Wallings RL, Tansey MG (2019). LRRK2 regulation of immune-pathways and inflammatory disease. Biochem Soc Trans, 47:1581-1595. 31769472 10.1042/BST20180463 PMC6925522

[78] Weindel CG, Bell SL, Vail KJ, West KO, Patrick KL, Watson RO (2020). LRRK2 maintains mitochondrial homeostasis and regulates innate immune responses to Mycobacterium tuberculosis. Elife, 9. 10.7554/eLife.51071 PMC7159881 32057291

[79] Main BS, Zhang M, Brody KM, Ayton S, Frugier T, Steer D, et al. (2016). Type-1 interferons contribute to the neuroinflammatory response and disease progression of the MPTP mouse model of Parkinson's disease. Glia, 64:1590-1604. 27404846 10.1002/glia.23028

[80] Sliter DA, Martinez J, Hao L, Chen X, Sun N, Fischer TD, et al. (2018). Parkin and PINK1 mitigate STING-induced inflammation. Nature, 561:258-262. 30135585 10.1038/s41586-018-0448-9 PMC7362342

[81] Crow YJ, Chase DS, Lowenstein Schmidt J, Szynkiewicz M, Forte GM, Gornall HL, et al. (2015). Characterization of human disease phenotypes associated with mutations in TREX1, RNASEH2A, RNASEH2B, RNASEH2C, SAMHD1, ADAR, and IFIH1. Am J Med Genet A, 167a:296-312. 25604658 10.1002/ajmg.a.36887 PMC4382202

[82] Crow YJ, Shetty J, Livingston JH (2020). Treatments in Aicardi-Goutières syndrome. Dev Med Child Neurol, 62:42-47. 31175662 10.1111/dmcn.14268

[83] Vizioli MG, Liu T, Miller KN, Robertson NA, Gilroy K, Lagnado AB, et al. (2020). Mitochondria-to-nucleus retrograde signaling drives formation of cytoplasmic chromatin and inflammation in senescence. Genes Dev, 34:428-445. 32001510 10.1101/gad.331272.119 PMC7050483

[84] Gehrke N, Mertens C, Zillinger T, Wenzel J, Bald T, Zahn S, et al. (2013). Oxidative damage of DNA confers resistance to cytosolic nuclease TREX1 degradation and potentiates STING-dependent immune sensing. Immunity, 39:482-495. 23993650 10.1016/j.immuni.2013.08.004

[85] Hemphill WO, Simpson SR, Liu M, Salsbury FR Jr., Hollis T, Grayson JM, et al. (2021). TREX1 as a Novel Immunotherapeutic Target. Front Immunol, 12:660184. 33868310 10.3389/fimmu.2021.660184 PMC8047136

[86] Stetson DB, Ko JS, Heidmann T, Medzhitov R (2008). Trex1 prevents cell-intrinsic initiation of autoimmunity. Cell, 134:587-598. 18724932 10.1016/j.cell.2008.06.032 PMC2626626

[87] Gray EE, Treuting PM, Woodward JJ, Stetson DB (2015). Cutting Edge: cGAS Is Required for Lethal Autoimmune Disease in the Trex1-Deficient Mouse Model of Aicardi-Goutières Syndrome. J Immunol, 195:1939-1943. 26223655 10.4049/jimmunol.1500969 PMC4546858

[88] Rice GI, Forte GM, Szynkiewicz M, Chase DS, Aeby A, Abdel-Hamid MS, et al. (2013). Assessment of interferon-related biomarkers in Aicardi-Goutières syndrome associated with mutations in TREX1, RNASEH2A, RNASEH2B, RNASEH2C, SAMHD1, and ADAR: a case-control study. Lancet Neurol, 12:1159-1169. 24183309 10.1016/S1474-4422(13)70258-8 PMC4349523

[89] Rothblum-Oviatt C, Wright J, Lefton-Greif MA, McGrath-Morrow SA, Crawford TO, Lederman HM (2016). Ataxia telangiectasia: a review. Orphanet J Rare Dis, 11:159. 27884168 10.1186/s13023-016-0543-7 PMC5123280

[90] Quek H, Luff J, Cheung K, Kozlov S, Gatei M, Lee CS, et al. (2017). Rats with a missense mutation in Atm display neuroinflammation and neurodegeneration subsequent to accumulation of cytosolic DNA following unrepaired DNA damage. J Leukoc Biol, 101:927-947. 27895165 10.1189/jlb.4VMA0716-316R

[91] Zaki-Dizaji M, Akrami SM, Azizi G, Abolhassani H, Aghamohammadi A (2018). Inflammation, a significant player of Ataxia-Telangiectasia pathogenesis? Inflamm Res, 67:559-570. 29582093 10.1007/s00011-018-1142-y

[92] Aguado J, Chaggar HK, Gómez-Inclán C, Shaker MR, Leeson HC, Mackay-Sim A, et al. (2021). Inhibition of the cGAS-STING pathway ameliorates the premature senescence hallmarks of Ataxia-Telangiectasia brain organoids. Aging Cell, 20: e13468. 34459078 10.1111/acel.13468 PMC8441292

[93] Quek H, Luff J, Cheung K, Kozlov S, Gatei M, Lee CS, et al. (2017). A rat model of ataxia-telangiectasia: evidence for a neurodegenerative phenotype. Hum Mol Genet, 26:109-123. 28007901 10.1093/hmg/ddw371

[94] Grivennikov SI, Greten FR, Karin M (2010). Immunity, inflammation, and cancer. Cell, 140:883-899. 20303878 10.1016/j.cell.2010.01.025 PMC2866629

[95] Nagata S, Hanayama R, Kawane K (2010). Autoimmunity and the clearance of dead cells. Cell, 140:619-630. 20211132 10.1016/j.cell.2010.02.014

[96] Trinchieri G (2012). Cancer and inflammation: an old intuition with rapidly evolving new concepts. Annu Rev Immunol, 30:677-706. 22224761 10.1146/annurev-immunol-020711-075008

[97] Ahn J, Xia T, Konno H, Konno K, Ruiz P, Barber GN (2014). Inflammation-driven carcinogenesis is mediated through STING. Nat Commun, 5:5166. 25300616 10.1038/ncomms6166 PMC4998973

[98] Barbie DA, Tamayo P, Boehm JS, Kim SY, Moody SE, Dunn IF, et al. (2009). Systematic RNA interference reveals that oncogenic KRAS-driven cancers require TBK1. Nature, 462:108-112. 19847166 10.1038/nature08460 PMC2783335

[99] Dekker E, Tanis PJ, Vleugels JLA, Kasi PM, Wallace MB (2019). Colorectal cancer. Lancet, 394:1467-1480. 31631858 10.1016/S0140-6736(19)32319-0

[100] Li J, Ma X, Chakravarti D, Shalapour S, DePinho RA (2021). Genetic and biological hallmarks of colorectal cancer. Genes Dev, 35:787-820. 34074695 10.1101/gad.348226.120 PMC8168558

[101] Xia T, Konno H, Ahn J, Barber GN (2016). Deregulation of STING Signaling in Colorectal Carcinoma Constrains DNA Damage Responses and Correlates With Tumorigenesis. Cell Rep, 14:282-297. 26748708 10.1016/j.celrep.2015.12.029 PMC4845097

[102] Zhong G, Peng C, Chen Y, Li J, Yang R, Wu M, et al. (2018). Expression of STING and PD-L1 in colorectal cancer and their correlation with clinical prognosis. Int J Clin Exp Pathol, 11:1256-1264. 31938220 PMC6958181

[103] Corrales L, Glickman LH, McWhirter SM, Kanne DB, Sivick KE, Katibah GE, et al. (2015). Direct Activation of STING in the Tumor Microenvironment Leads to Potent and Systemic Tumor Regression and Immunity. Cell Rep, 11:1018-1030. 25959818 10.1016/j.celrep.2015.04.031 PMC4440852

[104] Chon HJ, Kim H, Noh JH, Yang H, Lee WS, Kong SJ, et al. (2019). STING signaling is a potential immunotherapeutic target in colorectal cancer. J Cancer, 10:4932-4938. 31598165 10.7150/jca.32806 PMC6775531

[105] Zhu Q, Man SM, Gurung P, Liu Z, Vogel P, Lamkanfi M, et al. (2014). Cutting edge: STING mediates protection against colorectal tumorigenesis by governing the magnitude of intestinal inflammation. J Immunol, 193:4779-4782. 25320273 10.4049/jimmunol.1402051 PMC4308418

[106] Wei B, Xu L, Guo W, Wang Y, Wu J, Li X, et al. (2021). SHP2-Mediated Inhibition of DNA Repair Contributes to cGAS-STING Activation and Chemotherapeutic Sensitivity in Colon Cancer. Cancer Res, 81:3215-3228. 33820798 10.1158/0008-5472.CAN-20-3738

[107] Reisländer T, Groelly FJ, Tarsounas M (2020). DNA Damage and Cancer Immunotherapy: A STING in the Tale. Mol Cell, 80:21-28. 32810436 10.1016/j.molcel.2020.07.026

[108] Rastrelli M, Tropea S, Rossi CR, Alaibac M (2014). Melanoma: epidemiology, risk factors, pathogenesis, diagnosis and classification. In Vivo, 28:1005-1011. 25398793

[109] MacKie RM, Hauschild A, Eggermont AM (2009). Epidemiology of invasive cutaneous melanoma. Ann Oncol, 20 Suppl 6:vi1-7. 19617292 10.1093/annonc/mdp252 PMC2712590

[110] Markovic SN, Erickson LA, Rao RD, Weenig RH, Pockaj BA, Bardia A, et al. (2007). Malignant melanoma in the 21st century, part 1: epidemiology, risk factors, screening, prevention, and diagnosis. Mayo Clin Proc, 82:364-380. 17352373 10.4065/82.3.364

[111] Takashima K, Takeda Y, Oshiumi H, Shime H, Okabe M, Ikawa M, et al. (2016). STING in tumor and host cells cooperatively work for NK cell-mediated tumor growth retardation. Biochem Biophys Res Commun, 478:1764-1771. 27608599 10.1016/j.bbrc.2016.09.021

[112] Kim H, Kim H, Feng Y, Li Y, Tamiya H, Tocci S, et al. (2020). PRMT5 control of cGAS/STING and NLRC5 pathways defines melanoma response to antitumor immunity. Sci Transl Med, 12. 10.1126/scitranslmed.aaz5683 PMC7508354 32641491

[113] Ma Z, Xiong Q, Xia H, Liu W, Dai S, Cai S, et al. (2021). Carboplatin activates the cGAS-STING pathway by upregulating the TREX-1 (three prime repair exonuclease 1) expression in human melanoma. Bioengineered, 12:6448-6458. 34519260 10.1080/21655979.2021.1972198 PMC8806763

[114] Siegel RL, Miller KD, Fuchs HE, Jemal A (2021). Cancer Statistics, 2021. CA Cancer J Clin, 71:7-33. 33433946 10.3322/caac.21654

[115] Parkes EE, Walker SM, Taggart LE, McCabe N, Knight LA, Wilkinson R, et al. (2017). Activation of STING-Dependent Innate Immune Signaling By S-Phase-Specific DNA Damage in Breast Cancer. J Natl Cancer Inst, 109. 10.1093/jnci/djw199 PMC5441301 27707838

[116] Liu LC, Shen YC, Wang YL, Wu WR, Chang LC, Chen YH, et al. (2022). Growth-promoting function of the cGAS-STING pathway in triple-negative breast cancer cells. Front Oncol, 12:851795. 35992877 10.3389/fonc.2022.851795 PMC9385397

[117] Bakhoum SF, Ngo B, Laughney AM, Cavallo JA, Murphy CJ, Ly P, et al. (2018). Chromosomal instability drives metastasis through a cytosolic DNA response. Nature, 553:467-472. 29342134 10.1038/nature25432 PMC5785464

[118] Li J, Duran MA, Dhanota N, Chatila WK, Bettigole SE, Kwon J, et al. (2021). Metastasis and Immune Evasion from Extracellular cGAMP Hydrolysis. Cancer Discov, 11:1212-1227. 33372007 10.1158/2159-8290.CD-20-0387 PMC8102348

[119] Cogan D, Bakhoum SF (2020). Re-awakening Innate Immune Signaling in Cancer: The Development of Highly Potent ENPP1 Inhibitors. Cell Chem Biol, 27:1327-1328. 33217310 10.1016/j.chembiol.2020.11.001 PMC8221070

[120] Chen Q, Boire A, Jin X, Valiente M, Er EE, Lopez-Soto A, et al. (2016). Carcinoma-astrocyte gap junctions promote brain metastasis by cGAMP transfer. Nature, 533:493-498. 27225120 10.1038/nature18268 PMC5021195

[121] Hong C, Schubert M, Tijhuis AE, Requesens M, Roorda M, van den Brink A, et al. (2022). cGAS-STING drives the IL-6-dependent survival of chromosomally instable cancers. Nature, 607:366-373. 35705809 10.1038/s41586-022-04847-2

[122] Hu Y, Manasrah BK, McGregor SM, Lera RF, Norman RX, Tucker JB, et al. (2021). Paclitaxel Induces Micronucleation and Activates Pro-Inflammatory cGAS-STING Signaling in Triple-Negative Breast Cancer. Mol Cancer Ther, 20:2553-2567. 34583980 10.1158/1535-7163.MCT-21-0195 PMC8643310

[123] Sung H, Ferlay J, Siegel RL, Laversanne M, Soerjomataram I, Jemal A, et al. (2021). Global Cancer Statistics 2020: GLOBOCAN Estimates of Incidence and Mortality Worldwide for 36 Cancers in 185 Countries. CA Cancer J Clin, 71:209-249. 33538338 10.3322/caac.21660

[124] Rebello RJ, Oing C, Knudsen KE, Loeb S, Johnson DC, Reiter RE, et al. (2021). Prostate cancer. Nat Rev Dis Primers, 7:9. 33542230 10.1038/s41572-020-00243-0

[125] Feng D, Shi X, You J, Xiong Q, Zhu W, Wei Q, et al. (2022). A cellular senescence-related gene prognostic index for biochemical recurrence and drug resistance in patients with prostate cancer. Am J Cancer Res, 12:3811-3828. 36119834 PMC9441995

[126] Ho SS, Zhang WY, Tan NY, Khatoo M, Suter MA, Tripathi S, et al. (2016). The DNA Structure-Specific Endonuclease MUS81 Mediates DNA Sensor STING-Dependent Host Rejection of Prostate Cancer Cells. Immunity, 44:1177-1189. 27178469 10.1016/j.immuni.2016.04.010

[127] Suter MA, Tan NY, Thiam CH, Khatoo M, MacAry PA, Angeli V, et al. (2021). cGAS-STING cytosolic DNA sensing pathway is suppressed by JAK2-STAT3 in tumor cells. Sci Rep, 11:7243. 33790360 10.1038/s41598-021-86644-x PMC8012641

[128] Radej S, Szewc M, Maciejewski R (2022). Prostate Infiltration by Treg and Th17 Cells as an Immune Response to Propionibacterium acnes Infection in the Course of Benign Prostatic Hyperplasia and Prostate Cancer. Int J Mol Sci, 23. 36012113 10.3390/ijms23168849 PMC9408129

[129] Fischer K, Tschismarov R, Pilz A, Straubinger S, Carotta S, McDowell A, et al. (2020). Cutibacterium acnes Infection Induces Type I Interferon Synthesis Through the cGAS-STING Pathway. Front Immunol, 11:571334. 33178195 10.3389/fimmu.2020.571334 PMC7593769

[130] Hsu SC, Chen CL, Cheng ML, Chu CY, Changou CA, Yu YL, et al. (2021). Arginine starvation elicits chromatin leakage and cGAS-STING activation via epigenetic silencing of metabolic and DNA-repair genes. Theranostics, 11:7527-7545. 34158865 10.7150/thno.54695 PMC8210599

[131] Ma Z, Zhang W, Dong B, Xin Z, Ji Y, Su R, et al. (2022). Docetaxel remodels prostate cancer immune microenvironment and enhances checkpoint inhibitor-based immunotherapy. Theranostics, 12:4965-4979. 35836810 10.7150/thno.73152 PMC9274752

[132] Jackson SP, Bartek J (2009). The DNA-damage response in human biology and disease. Nature, 461:1071-1078. 19847258 10.1038/nature08467 PMC2906700

[133] Li T, Chen ZJ (2018). The cGAS-cGAMP-STING pathway connects DNA damage to inflammation, senescence, and cancer. J Exp Med, 215:1287-1299. 29622565 10.1084/jem.20180139 PMC5940270

[134] Liu H, Zhang H, Wu X, Ma D, Wu J, Wang L, et al. (2018). Nuclear cGAS suppresses DNA repair and promotes tumorigenesis. Nature, 563:131-136. 30356214 10.1038/s41586-018-0629-6

[135] Roehrborn CG (2008). Pathology of benign prostatic hyperplasia. Int J Impot Res, 20 Suppl 3: S11-18. 10.1038/ijir.2008.55 19002119

[136] Choi J, Shendrik I, Peacocke M, Peehl D, Buttyan R, Ikeguchi EF, et al. (2000). Expression of senescence-associated beta-galactosidase in enlarged prostates from men with benign prostatic hyperplasia. Urology, 56:160-166. 10869659 10.1016/s0090-4295(00)00538-0

[137] He S, Sharpless NE (2017). Senescence in Health and Disease. Cell, 169:1000-1011. 28575665 10.1016/j.cell.2017.05.015 PMC5643029

[138] Loo TM, Miyata K, Tanaka Y, Takahashi A (2020). Cellular senescence and senescence-associated secretory phenotype via the cGAS-STING signaling pathway in cancer. Cancer Sci, 111:304-311. 31799772 10.1111/cas.14266 PMC7004529

[139] Liu ZS, Cai H, Xue W, Wang M, Xia T, Li WJ, et al. (2019). G3BP1 promotes DNA binding and activation of cGAS. Nat Immunol, 20:18-28. 30510222 10.1038/s41590-018-0262-4 PMC8276115

[140] Dai J, Huang YJ, He X, Zhao M, Wang X, Liu ZS, et al. (2019). Acetylation Blocks cGAS Activity and Inhibits Self-DNA-Induced Autoimmunity. Cell, 176:1447-1460.e1414. 30799039 10.1016/j.cell.2019.01.016 PMC8274936

[141] Vincent J, Adura C, Gao P, Luz A, Lama L, Asano Y, et al. (2017). Small molecule inhibition of cGAS reduces interferon expression in primary macrophages from autoimmune mice. Nat Commun, 8:750. 28963528 10.1038/s41467-017-00833-9 PMC5622107

[142] Lama L, Adura C, Xie W, Tomita D, Kamei T, Kuryavyi V, et al. (2019). Development of human cGAS-specific small-molecule inhibitors for repression of dsDNA-triggered interferon expression. Nat Commun, 10:2261. 31113940 10.1038/s41467-019-08620-4 PMC6529454

[143] Chu L, Li C, Li Y, Yu Q, Yu H, Li C, et al. (2021). Perillaldehyde Inhibition of cGAS Reduces dsDNA-Induced Interferon Response. Front Immunol, 12:655637. 33968056 10.3389/fimmu.2021.655637 PMC8100446

[144] Tan J, Wu B, Chen T, Fan C, Zhao J, Xiong C, et al. (2021). Synthesis and Pharmacological Evaluation of Tetrahydro-γ-carboline Derivatives as Potent Anti-inflammatory Agents Targeting Cyclic GMP-AMP Synthase. J Med Chem, 64:7667-7690. 34044539 10.1021/acs.jmedchem.1c00398

[145] Li Q, Cao Y, Dang C, Han B, Han R, Ma H, et al. (2020). Inhibition of double-strand DNA-sensing cGAS ameliorates brain injury after ischemic stroke. EMBO Mol Med, 12: e11002. 32239625 10.15252/emmm.201911002 PMC7136961

[146] Siu T, Altman MD, Baltus GA, Childers M, Ellis JM, Gunaydin H, et al. (2019). Discovery of a Novel cGAMP Competitive Ligand of the Inactive Form of STING. ACS Med Chem Lett, 10:92-97. 30655953 10.1021/acsmedchemlett.8b00466 PMC6331172

[147] Li S, Hong Z, Wang Z, Li F, Mei J, Huang L, et al. (2018). The Cyclopeptide Astin C Specifically Inhibits the Innate Immune CDN Sensor STING. Cell Rep, 25:3405-3421.e3407. 30566866 10.1016/j.celrep.2018.11.097

[148] Hansen AL, Buchan GJ, Rühl M, Mukai K, Salvatore SR, Ogawa E, et al. (2018). Nitro-fatty acids are formed in response to virus infection and are potent inhibitors of STING palmitoylation and signaling. Proc Natl Acad Sci U S A, 115: E7768-e7775. 30061387 10.1073/pnas.1806239115 PMC6099880

[149] Haag SM, Gulen MF, Reymond L, Gibelin A, Abrami L, Decout A, et al. (2018). Targeting STING with covalent small-molecule inhibitors. Nature, 559:269-273. 29973723 10.1038/s41586-018-0287-8

[150] Hong Z, Mei J, Li C, Bai G, Maimaiti M, Hu H, et al. (2021). STING inhibitors target the cyclic dinucleotide binding pocket. Proc Natl Acad Sci U S A, 118. 10.1073/pnas.2105465118 PMC8214703 34099558

[151] Gao P, Ascano M, Zillinger T, Wang W, Dai P, Serganov AA, et al. (2013). Structure-function analysis of STING activation by c[G(2',5')pA(3',5')p] and targeting by antiviral DMXAA. Cell, 154:748-762. 23910378 10.1016/j.cell.2013.07.023 PMC4386733

[152] Cavlar T, Deimling T, Ablasser A, Hopfner KP, Hornung V (2013). Species-specific detection of the antiviral small-molecule compound CMA by STING. Embo j, 32:1440-1450. 23604073 10.1038/emboj.2013.86 PMC3655471

[153] Ager CR, Reilley MJ, Nicholas C, Bartkowiak T, Jaiswal AR, Curran MA (2017). Intratumoral STING Activation with T-cell Checkpoint Modulation Generates Systemic Antitumor Immunity. Cancer Immunol Res, 5:676-684. 28674082 10.1158/2326-6066.CIR-17-0049 PMC5547907

[154] Tang CH, Zundell JA, Ranatunga S, Lin C, Nefedova Y, Del Valle JR, et al. (2016). Agonist-Mediated Activation of STING Induces Apoptosis in Malignant B Cells. Cancer Res, 76:2137-2152. 26951929 10.1158/0008-5472.CAN-15-1885 PMC4873432

[155] Ramanjulu JM, Pesiridis GS, Yang J, Concha N, Singhaus R, Zhang SY, et al. (2018). Design of amidobenzimidazole STING receptor agonists with systemic activity. Nature, 564:439-443. 30405246 10.1038/s41586-018-0705-y

[156] Nakamura T, Sato T, Endo R, Sasaki S, Takahashi N, Sato Y, et al. (2021). STING agonist loaded lipid nanoparticles overcome anti-PD-1 resistance in melanoma lung metastasis via NK cell activation. J Immunother Cancer, 9. 10.1136/jitc-2021-002852 PMC8256839 34215690

[157] Pan BS, Perera SA, Piesvaux JA, Presland JP, Schroeder GK, Cumming JN, et al. (2020). An orally available non-nucleotide STING agonist with antitumor activity. Science, 369. 10.1126/science.aba6098 32820094

[158] Li S, Luo M, Wang Z, Feng Q, Wilhelm J, Wang X, et al. (2021). Prolonged activation of innate immune pathways by a polyvalent STING agonist. Nat Biomed Eng, 5:455-466. 33558734 10.1038/s41551-020-00675-9 PMC8126516

[159] C hin EN, Yu C, Vartabedian VF, Jia Y, Kumar M, Gamo AM, et al. (2020). Antitumor activity of a systemic STING-activating non-nucleotide cGAMP mimetic. Science, 369:993-999. 32820126 10.1126/science.abb4255

[160] Wang Y, Luo J, Alu A, Han X, Wei Y, Wei X (2020). cGAS-STING pathway in cancer biotherapy. Mol Cancer, 19:136. 32887628 10.1186/s12943-020-01247-w PMC7472700

